# Increased Network Inhibition in the Dentate Gyrus of Adult Neuroligin-4 Knock-Out Mice

**DOI:** 10.1523/ENEURO.0471-22.2023

**Published:** 2023-04-19

**Authors:** Julia Muellerleile, Matej Vnencak, Mohammad Valeed Ahmed Sethi, Tassilo Jungenitz, Stephan W. Schwarzacher, Peter Jedlicka

**Affiliations:** 1 Institute of Clinical Neuroanatomy, Neuroscience Center, Goethe University Frankfurt, 60590 Frankfurt am Main, Germany; 2 Faculty of Biosciences, Goethe University Frankfurt, 60439 Frankfurt am Main, Germany; 3 Faculty of Medicine, Justus-Liebig-University Giessen, 35392 Giessen, Germany

**Keywords:** dentate granule cell, in vivo electrophysiology, organotypic slice culture, paired-pulse inhibition, synaptic scaling, whole-cell patch-clamp recording

## Abstract

Loss-of-function mutations in neuroligin-4 (Nlgn4), a member of the neuroligin family of postsynaptic adhesion proteins, cause autism spectrum disorder in humans. Nlgn4 knockout (KO) in mice leads to social behavior deficits and complex alterations of synaptic inhibition or excitation, depending on the brain region. In the present work, we comprehensively analyzed synaptic function and plasticity at the cellular and network levels in hippocampal dentate gyrus of Nlgn4 KO mice. Compared with wild-type littermates, adult Nlgn4 KO mice exhibited increased paired-pulse inhibition of dentate granule cell population spikes, but no impairments in excitatory synaptic transmission or short-term and long-term plasticity *in vivo*. *In vitro* patch-clamp recordings in neonatal organotypic entorhino-hippocampal slice cultures from Nlgn4 KO and wild-type littermates revealed no significant differences in excitatory or inhibitory synaptic transmission, homeostatic synaptic plasticity, and passive electrotonic properties in dentate granule cells, suggesting that the increased inhibition *in vivo* is the result of altered network activity in the adult Nlgn4 KO. A comparison with prior studies on Nlgn 1–3 knock-out mice reveals that each of the four neuroligins exerts a characteristic effect on both intrinsic cellular and network activity in the dentate gyrus *in vivo*.

## Significance Statement

By linking the presynapse to the postsynapse, the neuroligin proteins play an important role in the stabilization and maturation of synapses in the CNS. Two of the four neuroligins that are shared between humans and mice, neuroligin-3 and neuroligin-4, are implicated in autism spectrum disorder in humans and autistic-like social and communication deficits in mice. However, the synaptic localization and function of neuroligin-4 in mice is not fully resolved. Here, we analyzed the contribution of neuroligin-4 to excitatory and inhibitory synaptic transmission in the hippocampal dentate gyrus and found that the excitation/inhibition ratio was decreased in adult neuroligin-4 knock-out mice, but unaltered in slice cultures prepared from neonatal neuroligin-4 knock-out mice.

## Introduction

Neuroligins 1–4 (Nlgn1–4) are transmembrane cell adhesion proteins that physically link the presynapse and postsynapse by binding to presynaptic neurexins (Südhof, 2017) and regulate synapse assembly and maturation via interactions with multiple postsynaptic scaffold and signaling proteins, such as PSD-95 (Irie et al., 1997; Bolliger et al., 2001), gephyrin, or collybistin (Poulopoulos et al., 2009). Nlgn4, arguably the most enigmatic of the four family members, is expressed at lower levels than the other neuroligins in mice (Varoqueaux et al., 2006), but plays an important role in the regulation of synaptic transmission in different brain regions (Hoon et al., 2011; Delattre et al., 2013; Hammer et al., 2015; Unichenko et al., 2018; Zhang et al., 2018). Strikingly, loss-of-function mutations in the human Nlgn4 ortholog NLGN4X cause autism spectrum disorder (ASD) in humans (Jamain et al., 2003; Yan et al., 2005; Kopp et al., 2021), and Nlgn4 knock-out (KO) mice exhibit ASD-like traits (Jamain et al., 2008; El-Kordi et al., 2013; Ju et al., 2014), establishing Nlgn4 KO mice as construct-valid and face-valid ASD models that can yield important insights into the pathomechanisms of ASD.

The synaptic localization and function of Nlgn4 in mice differs between brain regions. In retina and brainstem, Nlgn4 controls the assembly and function of glycinergic synapses (Hoon et al., 2011; Zhang et al., 2018), whereas it predominantly operates at GABAergic synapses in hippocampal area CA3 (Hammer et al., 2015) and at both excitatory and inhibitory synapses in the cortex (Delattre et al., 2013; Unichenko et al., 2018). Analyses of the human ortholog indicated a function of NLGN4X at excitatory synapses (Zhang et al., 2009; Bemben et al., 2015; Chanda et al., 2016; Marro et al., 2019; Cast et al., 2021). However, all corresponding studies involved conditions of NLGN4X overexpression, under which neuroligins are well known to lose any synapse-type specificity they may have *in vivo*.

The KO mouse model offers the key advantage of studying Nlgn4 function *in vivo* in defined brain regions that are involved in the cognitive and behavioral symptoms of ASD. One such region is the hippocampal dentate gyrus, which has been implicated in different forms of learning and memory, including social memory (Kesner, 2018), and serves an antiepileptogenic function by controlling the excitability of the hippocampus proper (Krook-Magnuson et al., 2015). The principal neurons of the dentate gyrus, the granule cells (GCs), are characterized by a low resting membrane potential and sparse firing activity, which is controlled by strong GABAergic inhibition. Since it was shown that Nlgn4 regulates perisomatic inhibition in CA3 pyramidal cells and Nlgn4 is expressed at similar levels throughout the hippocampus and the dentate gyrus (Hammer et al., 2015), we hypothesized that it might serve a similar function in dentate granule cells. Therefore, we analyzed the consequences of Nlgn4 KO on synaptic transmission and plasticity, neuronal excitability, and network inhibition in the dentate gyrus using *in vivo* field potential recordings.

Unexpectedly, we found that network inhibition was increased in the dentate gyrus of Nlgn4 KO mice, but excitatory synaptic transmission and Hebbian synaptic plasticity were unaltered. Whole-cell patch-clamp recordings in organotypic entorhino-hippocampal slice cultures revealed no significant differences in the intrinsic excitability of the Nlgn4 KO granule cells. Furthermore, excitatory and inhibitory synaptic transmission as well as the excitation/inhibition (E/I) ratio of individual granule cells were not affected by the absence of Nlgn4. These results suggest that the function of Nlgn4 in adult mice differs from its function in slice cultures from young mice, perhaps because of age-related changes in the expression level or compensatory mechanisms in the adult.

## Materials and Methods

### Animals

All animal procedures were performed in accordance with the Goethe University animal care committee regulations. Mice were housed in individually ventilated cages or in filter-top cages within a ventilated cabinet (Scantainer) at a constant temperature and a 12 h light/dark cycle (lights on at 7:00 A.M.) with food and water available *ad libitum*. Nlgn4 KO (RRID:MGI:3775814; Jamain et al., 2008) and wild-type (WT) littermates from heterozygote interbreedings were used in all experiments. Genotyping was conducted as previously described (El-Kordi et al., 2013). All experiments were conducted by researchers blind to the genotype of the mice.

### Surgery and *in vivo* electrophysiology

For the *in vivo* experiments, only male mice were used to exclude possibly confounding effects of the estrous cycle on synaptic transmission and plasticity (Spencer et al., 2008). The 8- to 12-week-old male mice were anesthetized via an intraperitoneal urethane injection (1.2 g/kg body weight in the initial dose, then 0.2–0.5 g/kg doses injected subcutaneously for maintenance) and placed in a stereotactic frame (Kopf) for the insertion of the stimulation and recording electrodes according to the coordinates from a mouse brain atlas (Franklin and Paxinos, 1997). Prilocaine hydrochloride with adrenaline (xylonest 1%, AstraZeneca) was applied for local anesthesia of the scalp. A bipolar stimulation electrode (tip separation, 0.5 mm; model NE-200, Rhodes Medical Instruments) was inserted into the angular bundle of the perforant path (coordinates: 3.7 mm posterior to bregma, 2.5 mm lateral to the midline, 1.8 mm below the brain surface), and a tungsten recording electrode (model TM33A10KT, World Precision Instruments) was positioned above the ipsilateral dentate gyrus (coordinates: 1.7 mm posterior to bregma, 1.0 mm lateral to the midline). The recording electrode was lowered in 0.05–0.1 mm increments until the suprapyramidal granule cell layer was reached, as determined by the waveform in response to perforant path stimulation using 500 μA/0.1 ms current pulses provided by a stimulus generator (model STG1004, Multichannel Systems). The field excitatory postsynaptic potentials (fEPSPs) were preamplified (P55 Preamplifier, Grass Technologies) and digitized at 10 kHz (Digidata 1440A, Molecular Devices) for visualization and offline analysis. The stimulation protocols were applied in the following order: increasing stimulation intensities from 30 to 800 μA for input–output measurements, low-intensity paired-pulse stimulation to elicit paired-pulse facilitation of the fEPSP, maximal and minimal intensity paired-pulse stimulation to elicit paired-pulse inhibition of the population spike, and theta-burst stimulation (TBS) for the induction of long-term potentiation (LTP). Before LTP induction, a 10 min baseline was recorded at a stimulation intensity set to elicit a 1–2 mV population spike. The stimulation intensity and duration were doubled during the TBS, which consisted of six series (separated by 20 s) of six trains (separated by 0.2 s) of six 400 Hz pulses. After TBS, evoked field potentials using the pre-TBS stimulation intensity were recorded for 1 h. At the end of the experiment, the deeply anesthetized mice were transcardially perfused with 4% (w/v) paraformaldehyde (PFA; Honeywell/Fluka), and the brains were stored at −20°C until further use.

### Preparation of organotypic entorhino-hippocampal slice cultures

Slice cultures were prepared from mice of either sex at postnatal day 4 (P4) or P5. Brains were rapidly removed, attached to a vibratome base plate with tissue glue (Histoacryl, B. Braun), and placed in preparation medium containing Gibco HEPES-buffered minimum essential medium (Thermo Fisher Scientific) containing Earle’s salts, 0.65% glucose, 0.1 mg/ml streptomycin, and 100 U/ml penicillin (all from Merck/Sigma-Aldrich); and 2 mm Gibco GlutaMAX (Thermo Fisher Scientific), adjusted to a pH between 7.3 and 7.4 with HCl (VWR) and NaOH. The 300-μm-thick horizontal sections were cut with a vibratome (model VT1000S, Leica) set to a low speed (0.13 mm/s) and high frequency (80–90 Hz). The hippocampus and the attached entorhinal cortex were dissected with sterile scalpels and carefully placed on a membrane filter insert (pore size, 0.4 μm; diameter, 30 mm; Millicell-CM, Millipore) in a six-well plate containing prewarmed incubation medium (1 ml/well). The incubation medium was identical to the preparation medium but was supplemented with 25% (v/v) heat-inactivated normal horse serum (Thermo Fisher Scientific) and buffered with 0.15% (v/v) sodium bicarbonate to maintain a pH of 7.3 in the presence of CO_2_. Slice cultures were maintained at 35°C in a humidified incubator (95% air, 5% CO_2_; Heraeus) for a minimum of 18 d before experimental manipulation, and the incubation medium was exchanged fully every 2–3 d. The day of preparation was considered 0 d *in vitro* (0 DIV).

### Tetrodotoxin treatment of mature organotypic entorhino-hippocampal slice cultures

Mature (≥18 DIV) slice cultures were treated with 2 μm tetrodotoxin (TTX; Alomone Labs) dissolved in water or pure water by pipetting 1 μl of the solution into the well. After 48 h, the slice cultures were used for patch-clamp recordings. TTX-treated slice cultures were placed in TTX-containing artificial cerebrospinal fluid (ACSF) when they were cut out of the filter insert, whereas vehicle-treated slice cultures were cut in normal ACSF.

### Electrophysiological recording of granule cells in organotypic entorhino-hippocampal slice cultures

Whole-cell patch-clamp recordings were obtained from dentate GCs at 20–26 DIV. The bath solution consisted of ACSF made up of the following (in mm): 126 NaCl, 2.5 KCl, 26 NaHCO_3_, 1.25 NaH_2_PO_4_, 2 CaCl_2_, 2 MgCl_2_ (all from Merck/Sigma-Aldrich); and 10 glucose (AppliChem). The pH was maintained with a mixture of 95% O_2_/5% CO_2_, and the bath temperature was set to 28–30°C. In most experiments, an intracellular solution was used consisting of the following (in mm): 126 potassium gluconate, 10 HEPES, 4 KCl, 4 Mg-ATP (all from Merck/Sigma-Aldrich), 0.3 Na_2_-GTP (Carl Roth), 10 Na_2_-phosphocreatine (Merck/Sigma-Aldrich) and 0.3% (w/v) biocytin (Cayman Chemical) adjusted to pH 7.25 with KOH. The osmolality was adjusted to ∼285 mOsm/kg with glucose. During miniature excitatory postsynaptic current (mEPSC) recording, 0.5 μm TTX, 10 μm d-APV, and 10 μm gabazine (all from Alomone Labs) were added to the bath solution. For recording spontaneous EPSCs (sEPSCs) and evoked EPSCs and inhibitory postsynaptic currents (IPSCs) from the same cell, a modified version of a previously published cesium gluconate-based intracellular solution (Le Prieult et al., 2017) was used which consisted of (in mM) 130 cesium gluconate (Hellobio), 10 HEPES, 10 EGTA, 2 MgCl_2_, and 2 Na_2_-ATP (all from Merck/Sigma-Aldrich); 0.4 -Na_2_-GTP (Carl Roth); 5 QX314-Cl (Alomone Labs); and 0.3% (w/v) biocytin (Cayman Chemical) adjusted to a pH of 7.25 and an osmolality of ∼285 mOsm/kg with CsOH and glucose, respectively. In these experiments, 20 μm d-APV was added to the bath solution to block NMDA receptors.

Patch pipettes were prepared from borosilicate glass capillaries (outer diameter, 1.5 mm; model GC150TF-10, Warner Instruments/Harvard Apparatus) pulled to a tip resistance of 3–5 MΩ using a horizontal puller (DMZ-Universal Electrode Puller, Zeitz). The dentate gyrus was identified by infrared-differential interference contrast videomicroscopy using an upright microscope (model Axioscope 2FS, ZEISS) equipped with a 40× water-immersion objective (Achroplan 0.8 numerical aperture, both from ZEISS) coupled to an infrared-sensitive CCD camera (Hamamatsu). Whole-cell patch-clamp recordings of granule cells of the suprapyramidal blade of the dentate gyrus were made with a Multiclamp 700B amplifier and a CV-7B headstage (Molecular Devices) at a holding potential of −70 mV except during experiments in which the cesium-based intracellular solution was used, in which sEPSCs and spontaneous IPSCs (sIPSCs) were recorded at −60 and 10 mV, respectively. Data were digitized at 10 kHz (Digidata 1440A, Molecular Devices). The resting membrane potential was measured immediately after break-in. Series resistance was monitored every 1–2 min, and recordings were discarded if the series resistance and leak current reached ≥30 MΩ or ≥100 pA, respectively.

The frequency–current (*F–I*) relationship was measured in current-clamp configuration. Square current pulses (duration, 1 s) were applied in 10 pA increments from −100 to 490 pA, with 1 s interpulse intervals. The input resistance was calculated from the linear fit of the voltage difference plotted against the current injection during the negative current steps (−100 to −10 pA). At the end of the experiment, the series resistance was measured again, and only those cells in which the series resistance remained <30 MΩ were included in the final analysis.

Evoked EPSCs and IPSCs were obtained by electrically stimulating the middle and outer molecular layer with a concentric bipolar electrode (model #CBAPC75, FHC) with a stimulus generator (stimulus duration, 1 ms; model STG1004, Multichannel Systems). For each cell, the stimulation intensity was adjusted to the minimum current intensity that elicited an EPSC (between 2 and 100 μA). Per cell, five to six responses were recorded consecutively with 15 s interstimulus intervals at −60 and 10 mV holding potential. The series resistance was measured after the completion of the stimulation protocol, and only those cells in which the series resistance remained <30 MΩ were included in the analysis.

### Staining and *post hoc* identification of granule cells

Following electrophysiological recording, slice cultures were fixed in solution containing 4% (w/v) PFA and 4% (w/v) sucrose in PBS 0.1 m, pH 7.4 (A0964, PanReac AppliChem, Thermo Fisher Scientific) for 1 h and 2% (w/v) PFA/30% (w/v) sucrose in PBS overnight. After washing in PBS, membranes were permeabilized and nonspecific binding was blocked using a solution of Triton X-100 (VWR) and normal goat serum (NGS; Thermo Fisher Scientific) in PBS. Biocytin-filled neurons were stained by incubation with Alexa Fluor 488-conjugated streptavidin (1:500 in PBS, 1% NGS, 0.2% Triton X-100; Thermo Fisher Scientific) for 1 h, and nuclei were subsequently stained with TO-PRO Iodide (1:5000 in PBS; Thermo Fisher Scientific) for 15 min. Slice cultures were mounted on glass slides with antifade mounting medium (Dako Fluorescence Mounting Medium, Agilent) and observed under a confocal microscope (model Eclipse C1si Laser-Scanning Microscope, Nikon). Stained neurons were *post hoc* identified as granule cells of the suprapyramidal blade of the dentate gyrus based on morphologic and anatomic criteria (identification of dendrites and/or axonal projections within the dentate gyrus).

### Data analysis and statistics

Data were analyzed with Clampfit 10.7 software (Molecular Devices), Excel 2016 (Microsoft), MATLAB version R2018b (MathWorks), and Python version 3.9 (Python Software Foundation; https://www.python.org/). MiniAnalysis software (version 6.0.7; Synaptosoft) was used to quantify the sEPSCs/mEPSCs and sIPSCs. Traces were low-pass filtered using an elliptic filter with a cutoff frequency of 1000 Hz. The threshold for event detection was set to 3 pA for EPSCs and 10 pA for IPSCs. A minimum of 100 events per recorded neuron were selected by an investigator blind to the genotype and experimental treatment. Frequency–current and action potential analyses were performed with custom MATLAB scripts. Spike frequency adaptation was quantified by the ratio of the first interspike interval (ISI) to the last ISI in a spike train.

All statistical analyses were performed with Prism 8.0 software (GraphPad Software). Mean values were tested for normality using the D’Agostino–Pearson test. If the data were normally distributed, parametric statistical tests were used to compare group values. A two-way (repeated-measures) ANOVA or a mixed model (if there were missing values) was used to test for significant differences between groups that differed in more than one variable, and the Bonferroni correction for multiple comparisons was applied to all post-tests. The mixed model implemented in GraphPad Prism 8.0 uses a compound symmetry covariance matrix and is fit using a restricted maximum likelihood. The results can be interpreted like a repeated-measures ANOVA (Motulsky, 2022). Normality was assessed with quantile–quantile plots. The α level was set to 0.05, and only two-tailed *p*-values were computed. A Boltzmann sigmoidal equation was used to fit the EPSP-spike and paired-pulse inhibition data. The bottom was set to zero, and only fits with a goodness-of-fit (*R*^2^) value >0.8 were included in the final analysis. All group values are given as the mean ± SEM along with the 95% confidence interval (CI). For each statistical comparison, the difference between the means (or the median) and the 95% confidence interval of the difference are reported in the statistical table (see Table 2).

## Results

### Unchanged excitatory synaptic transmission in the dentate gyrus of Nlgn4 KO mice

**Figure 1.**
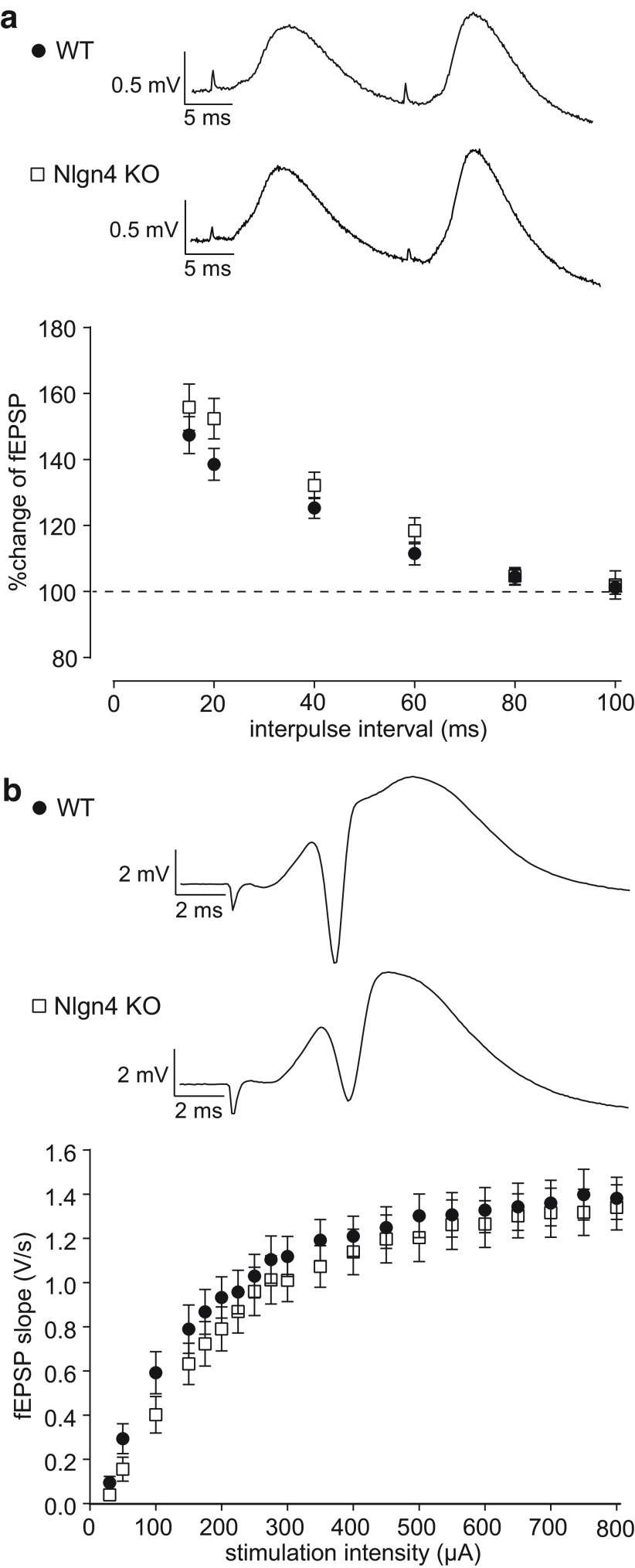
Unchanged excitatory synaptic transmission in the dentate gyrus of Nlgn4 KO mice *in vivo*. ***a***, Paired-pulse facilitation of the granule cell fEPSP at different interpulse intervals was not significantly different in KO mice (WT mice, *N *=* *17; KO mice, *N* = 16; two-way repeated-measures ANOVA: *p *<* *0.0001 for interpulse interval, *p *=* *0.18 for genotype, *p *=* *0.29 for interaction) as illustrated by representative traces from one WT and one KO mouse at 20 ms interpulse intervals (top). ***b***, No significant differences in the postsynaptic strength measured by the fEPSP slope (WT mice, *N *=* *17; KO mice, *N* = 16; two-way repeated-measures ANOVA: *p *<* *0.0001 for stimulation intensity, *p *=* *0.46 for genotype, *p *=* *0.96 for interaction), traces show the evoked fEPSP in response to a 700 μA stimulation pulse from one WT and one KO mouse (top).

We first tested whether Nlgn4 plays a role at excitatory synapses in the dentate gyrus of adult mice. Immunostaining for Nlgn4 in the hippocampus has been unsuccessful, possibly because of post-translational modifications of the protein, so its synaptic localization has to be deduced from electrophysiological measurements in KO mice (Hammer et al., 2015). We stimulated the perforant path and measured evoked field potentials in the dentate gyrus of WT and Nlgn4 KO mice to probe excitatory synaptic transmission from the perforant path to granule cells. Presynaptic short-term plasticity was quantified by the facilitation of the second fEPSP in response to double-pulse stimulation at increasing interpulse intervals. This analysis revealed no significant differences between the WT and KO mice (Fig. 1*a*; *N *=* *17 WT; *N* = 16 KO mice; two-way repeated-measures ANOVA: *p *<* *0.0001 for interpulse interval, *p *=* *0.18 for genotype, *p *=* *0.29 for interaction)^a^. To examine postsynaptic function, we measured the slope of the fEPSP in response to different stimulation intensities and likewise found no differences between groups (Fig. 1*b*; two-way repeated-measures ANOVA: *p *<* *0.0001 for stimulation intensity, *p *=* *0.46 for genotype, *p *=* *0.96 for interaction)^b^. Therefore, it appears that Nlgn4 is not required for basal excitatory synaptic transmission onto granule cells in adult mice.

### Unaltered induction of long-term potentiation at perforant path–granule cell synapses in Nlgn4 KO mice

**Figure 2.**
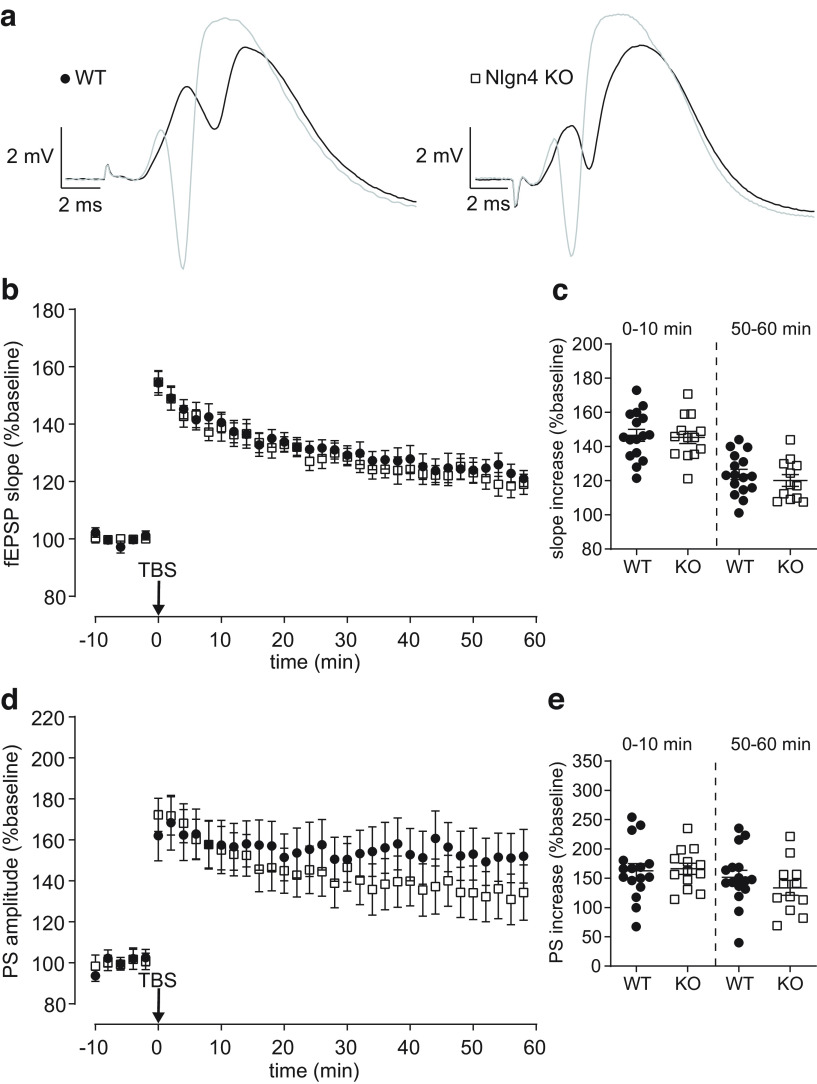
Unaltered long-term potentiation at perforant path–granule cell synapses in Nlgn4 KO mice. ***a***, Averaged traces showing the evoked fEPSP during the last 2 min of the baseline (black) and during the first 2 min following TBS (gray) from one WT and one KO mouse. ***b***, ***c***, The time course of the fEPSP slope expressed relative to the pre-TBS baseline (***b***) and the quantification of the slope increase (unpaired *t* test with Welch’s correction; 0–10 min: WT mice, *N *=* *16; KO mice, *N* = 13; *p *=* *0.81; 50–60 min: WT mice, *N *=* *16; KO mice, *N* = 12; *p *=* *0.44; ***c***) revealed a similar degree of potentiation in WT and KO mice. ***d***, ***e***, The time course of the population spike amplitude expressed relative to the pre-TBS baseline (***d***) and the quantification of the spike increase (unpaired *t* test with Welch’s correction; 0–10 min: WT mice, *N *=* *16; KO mice, *N *=* *13; *p *=* *0.83; 50–60 min: WT mice, *N *=* *16; and KO mice, *N *=* *12; *p *=* *0.32; ***e***) also showed no significant differences between WT and KO mice.

To test whether Nlgn4 is involved in Hebbian synaptic plasticity at perforant path–granule cell synapses, we induced LTP using a strong TBS protocol and compared the potentiation of the fEPSP slope in WT and Nlgn4 KO mice (Fig. 2*a*, examples). There were no significant differences in the increase of the fEPSP slope during the initial phase (Fig. 2*b*,*c*; WT: 146.5 ± 3.5%; 95% CI, 139.1, 154.0; *N *=* *16; KO: 145.3 ± 3.6%; 95% CI, 137.6, 153.1; *N *=* *13; unpaired *t* test with Welch’s correction, *p *=* *0.81) or during the final 10 min of recording (WT: 123.7 ± 3.0%, 95% CI, 117.2, 130.1; *N *=* *16; KO: 120.1 ± 3.4%; 95% CI, 112.6, 127.6; *N *=* *12; unpaired *t* test with Welch’s correction, *p *=* *0.44)^c^, indicating that Hebbian synaptic plasticity is not affected by the loss of Nlgn4.

We also analyzed the potentiation of the population spike, a second component of LTP that is not dependent on the degree of synaptic potentiation (Taube and Schwartzkroin, 1988). Approximately 50% of the increase in the population spike amplitude induced by TBS are because of nonsynaptic changes (i.e., changes in the intrinsic excitability of granule cells; Lopez-Rojas et al., 2016). Therefore, a reduction in the spike potentiation could reflect a decrease in the intrinsic excitability. However, the increase in the population spike following TBS was very similar in both groups during the initial phase (Fig. 2*d*,*e*; WT: 162.7 ± 12.3%; 95% CI, 136.4, 188.9; *N *=* *16; KO: 166.0 ± 9.4%; 95% CI, 145.4, 186.5; *N *=* *13; unpaired *t* test with Welch’s correction, *p *=* *0.83) and was only slightly lower in the Nlgn4 KO mice during the final phase (WT: 151.4 ± 12.0%; 95% CI, 125.8, 177.1; *N *=* *16; KO: 133.6 ± 12.8%; 95% CI, 105.4, 161.7; *N *=* *12; unpaired *t* test with Welch’s correction, *p *=* *0.32)^d^. Thus, we concluded that the TBS-induced potentiation of the population spike is not severely affected by the loss of Nlgn4.

### No significant difference in the granule cell excitability in Nlgn4 KO mice

**Figure 3.**
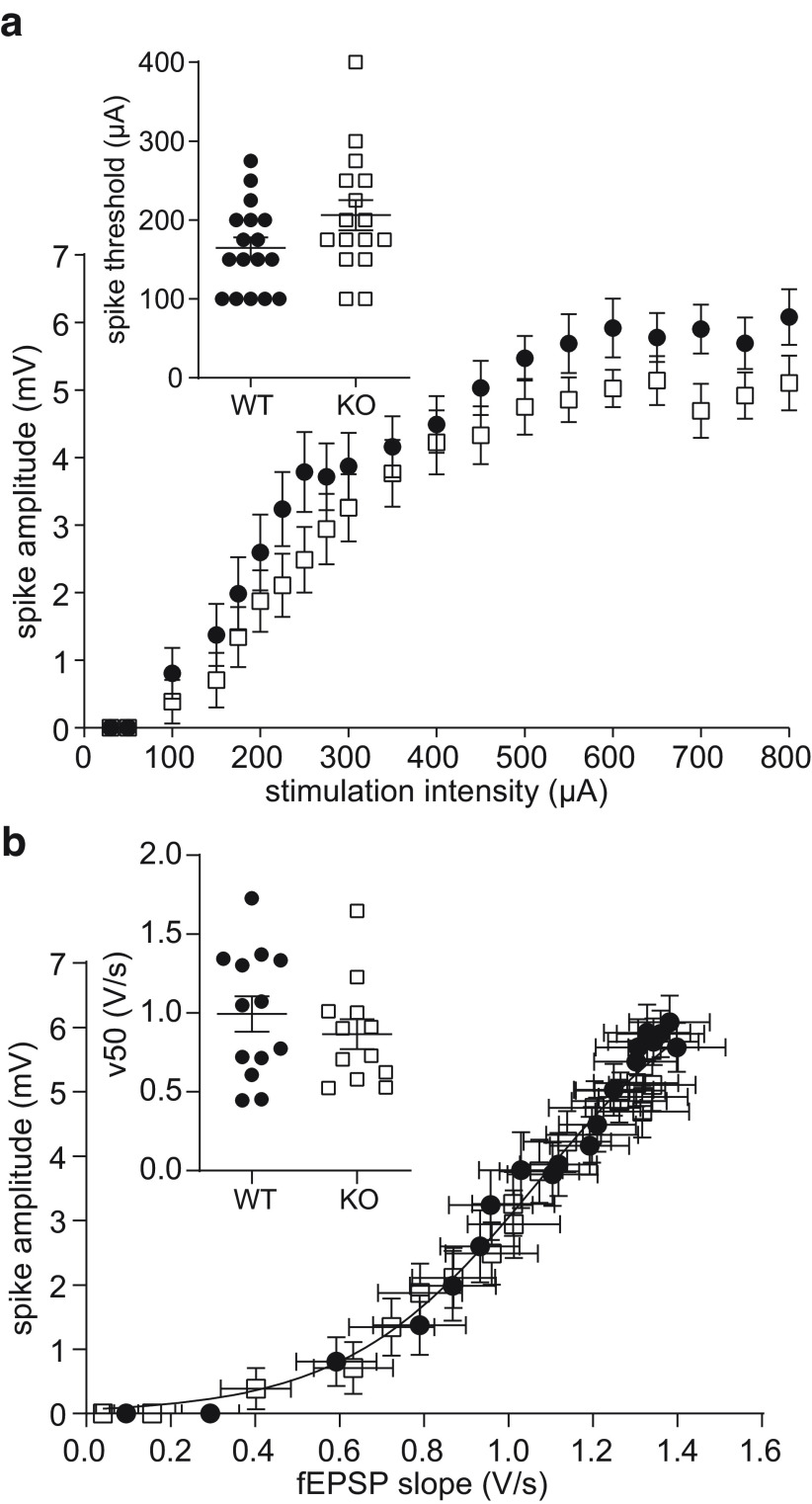
Slight differences in granule cell excitability, but unaltered EPSP–spike coupling, in Nlgn4 KO mice. ***a***, Granule cell excitability was assessed by the amplitude of the population spike (WT mice, *N *=* *17; KO mice, *N *=* *16; two-way repeated-measures ANOVA: *p *<* *0.0001 for stimulation intensity, *p *=* *0.15 for genotype, *p *=* *0.68 for interaction) at increasing stimulation intensities, and the onset of the population spike occurred at slightly higher stimulation intensities in Nlgn4 KO mice compared with WT mice (inset, unpaired *t* test with Welch’s correction, *p *=* *0.087). ***b***, The EPSP–spike relationship fitted by a Boltzmann sigmoidal function revealed no differences, as quantified by the v50 parameter of the fit function (inset: WT mice, *N *=* *13; KO mice, *N *=* *12; unpaired *t* test with Welch’s correction, *p *=* *0.40; Fig. 1*b*, example traces).

As a measure of the basal granule cell excitability, we compared the amplitude of the population spike in response to different stimulation intensities between WT and KO mice. While the population spike amplitudes were slightly reduced in the Nlgn4 KO mice, this difference was not significant (Fig. 3*a*; *N *=* *17 WT mice, *N* = 16 KO mice; two-way repeated-measures ANOVA: *p *<* *0.0001 for stimulation intensity, *p *=* *0.15 for genotype, *p *=* *0.68 for interaction)^e^. The stimulation intensity at which the first population spike appeared was slightly, but not significantly, increased in the Nlgn4 KO mice (206.3 ± 19.16 μA; 95% CI, 165.4, 247.1) compared with their WT littermates (164.7 ± 13.40 μA; 95% CI, 136.3, 193.1; Fig. 3*a*, inset; unpaired *t* test with Welch’s correction, *p *=* *0.087)^f^. We also examined the EPSP–spike curves (i.e., the relationship between fEPSP slope and population spike amplitude at each stimulation intensity) and fitted these with a Boltzmann sigmoidal function. The mean EPSP–spike curves could be fitted using the same parameters (Fig. 3*b*; extra sum-of-squares *F* test, *p *=* *0.14), and the comparison of the x-value (in this case, the fEPSP slope) at which 50% of the maximum y-value (here, the population spike amplitude) was attained (v50 parameter) yielded no significant difference between WT and Nlgn4 KO (Fig. 3*b*, inset; *N *=* *13 WT mice, *N* = 12 KO mice; unpaired *t* test with Welch’s correction, *p *=* *0.40)^g^.

### Altered network inhibition in the dentate gyrus of Nlgn4 KO mice

**Figure 4.**
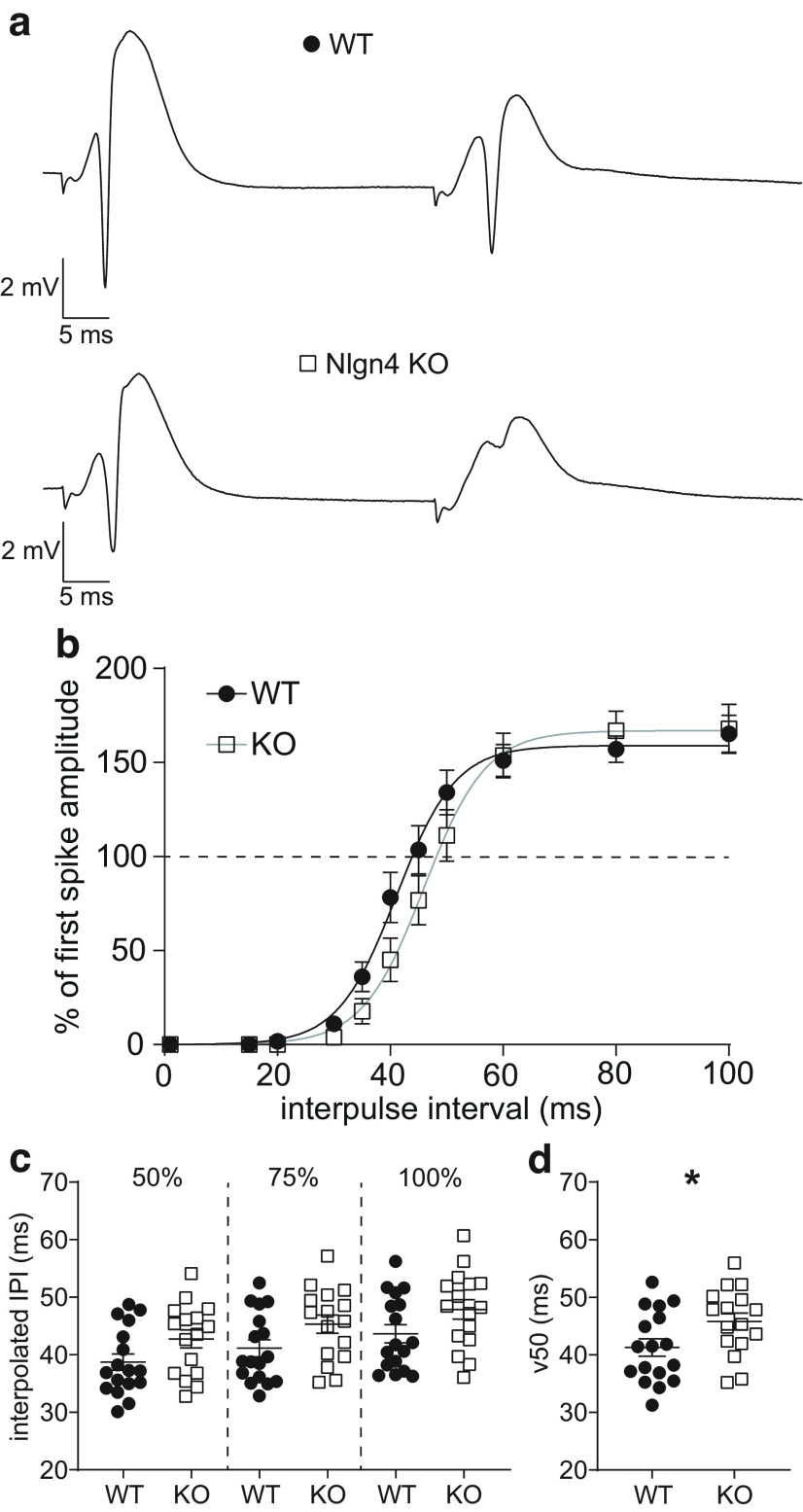
Increased network inhibition in the dentate gyrus of Nlgn4 KO mice. ***a***, Evoked fEPSPs from a WT and a KO mouse in response to 800 μA double-pulse stimulation delivered at 40 ms interpulse intervals. ***b***, Paired-pulse inhibition of the granule cell population spike was measured in response to 800 μA at different interpulse intervals (WT, *N *=* *17; KO mice, *N *= 16; two-way repeated-measures ANOVA: *p *=* *0.30 for genotype, *p *<* *0.0001 for interpulse interval, *p *=* *0.029 for interaction). ***c***, Interpolated interpulse intervals at 50% (unpaired *t* test with Welch’s correction, *p *=* *0.064), 75% (unpaired *t* test with Welch’s correction, *p *=* *0.064), and 100% (unpaired *t* test with Welch’s correction, *p *=* *0.076) relative amplitude of the second population spike. ***d***, The v50 parameter of the Boltzmann sigmoidal function used to fit the paired-pulse inhibition curve was higher in Nlgn4 KO mice (unpaired *t* test with Welch’s correction, *p *=* *0.040). See Extended Data Figure 4-1 for paired-pulse inhibition in response to the minimal stimulation intensity to evoke a population spike.

While excitatory synaptic transmission and plasticity appeared to be unaltered in the Nlgn4 KO, we suspected that inhibitory synaptic transmission from interneurons onto granule cells might be decreased, as reported previously for CA3 pyramidal cells (Hammer et al., 2015). Therefore, we used a paired-pulse protocol to examine feedback and feedforward inhibition of the granule cell population spike (Fig. 4*a*), which is mediated by local interneurons (Sloviter, 1991). Surprisingly, we observed what appeared to be an increase in network inhibition in Nlgn4 KO mice, indicated by a slight rightward shift of the curve relating the level of inhibition to the interpulse interval when using the maximal stimulation intensity of 800 μA (Fig. 4*b*)^h^. While the two-way repeated-measures ANOVA indicated no significant difference between genotypes (17 WT mice and 16 KO mice; *p *<* *0.0001 for interpulse interval, *p *=* *0.30 for genotype, *p *=* *0.029 for interaction), the interaction effect was significant, and the data were best fit by different curves (nonlinear least squares regression, Boltzmann sigmoidal function, *p *=* *0.0004). To quantify the degree of inhibition, the early portion of the curve (until a 100 ms interpulse interval) was fitted by a Boltzmann sigmoidal function and the corresponding interpulse interval at which the amplitude of the second population spike reached 50%, 75%, and 100% of the first population spike amplitude was interpolated. The mean interpulse intervals indicated a trend toward greater inhibition in the Nlgn4 KO (50%: WT = 38.71 ± 1.42 ms; 95% CI, 35.70, 41.72; *N *=* *17; KO = 42.75 ± 1.55 ms; 95% CI, 39.45, 46.05; *N *=* *16; unpaired *t* test with Welch’s correction, *p *=* *0.064; 75%: WT = 41.14 ± 1.48 ms; 95% CI, 38.01, 44.28; *N *=* *17; KO = 45.32 ± 1.59 ms; 95% CI, 41.93, 48.70; *N *=* *16; unpaired *t* test with Welch’s correction, *p *=* *0.064; 100%: WT = 43.64 ± 1.57 ms; 95% CI, 40.32, 46.97; *N *=* *17; KO = 47.87 ± 1.69 ms; 95% CI, 44.27, 51.46; *N *=* *16; unpaired *t* test with Welch’s correction, *p *=* *0.076; Fig. 4*c*)^i^ and the v50 parameter of the Boltzmann-fitted curve was significantly higher in KO mice (Fig. 4*d*; WT = 41.28 ± 1.50 ms; 95% CI, 38.08, 44.48; KO = 45.79 ± 1.46 ms; 95% CI, 42.68, 48.91; unpaired *t* test with Welch’s correction, *p *=* *0.040)^j^.

10.1523/ENEURO.0471-22.2023.f4-1 Figure 4-1 No difference in network inhibition evoked by minimal stimulation of the granule cell population spike. ***a***, Evoked fEPSPs from a WT and a KO mouse in response to minimal stimulation intensities (250 μA for WT, 170 μA for KO) to elicit a population spike, 40 ms interpulse intervals. ***b***, Paired-pulse inhibition of the granule cell population spike was measured in response to minimal stimulation at different interpulse intervals (*N *=* *12 mice per group, two-way repeated-measures ANOVA, *p *=* *0.99 for genotype, *p *>* *0.99 for interaction) and fit by a Boltzmann sigmoidal function. ***c***, Interpolated interpulse intervals at 50% (Mann−Whitney test, *p *=* *0.84), 75% (Mann−Whitney test, *p *=* *0.93), and 100% (Mann−Whitney test, *p *>* *0.99) relative amplitude of the second population spike. ***d***, The v50 parameter of the Boltzmann sigmoidal function used to fit the paired-pulse inhibition curve was not significantly different (Mann−Whitney test, *p *=* *0.71) Download Figure 4-1, EPS file.

Using the minimal stimulation intensity that elicited a spike (Extended Data Fig. 4-1*a*, examples), there were no significant differences in the paired-pulse inhibition curve (*N *=* *12 per group; two-way ANOVA: *p *<* *0.0001 for interpulse interval, *p *=* *0.99 for genotype, *p *>* *0.99 for interaction; nonlinear least-squares regression, Boltzmann sigmoidal function, *p *=* *0.84; Extended Data Fig. 4-1*b*)^k^, the interpolated interpulse intervals (50%: WT = 42.38 ± 3.60 ms; 95% CI, 34.46, 50.31; *N *=* *12; KO = 42.78 ± 2.20 ms; 95% CI, 37.94, 47.62; *N *=* *16; Mann–Whitney test, *p *=* *0.84; 75%: WT = 45.81 ± 3.85 ms; 95% CI, 37.33, 54.28; *N *=* *17; KO = 45.58 ± 2.43 ms; 95% CI, 40.23, 50.93]; *N *=* *16; Mann–Whitney test, *p *=* *0.93; 100%: WT = 47.38 ± 4.33 ms; 95% CI, 37.74, 57.02; *N *=* *17; KO = 46.25 ± 2.35 ms; 95% CI, 41.01, 51.48; *N *=* *16; Mann–Whitney test, *p *>* *0.99; Extended Data Fig. 4-1*c*)^l^, and the v50 parameter (WT: 48.59 ± 3.18 ms; 95% CI, 41.60, 55.59; *N *=* *12; KO: 48.61 ± 1.97 ms; 95% CI, 44.27, 52.95; *n *=* *12; Mann–Whitney test, *p *=* *0.71; Extended Data Fig. 4-1*d*)^m^ between WT and KO mice, indicating that differences in the level of network inhibition only occurred when the granule cells were maximally stimulated.

### Unaltered passive electrotonic properties and trend toward higher excitability in dentate granule cells Nlgn4 KO slice cultures

**Figure 5.**
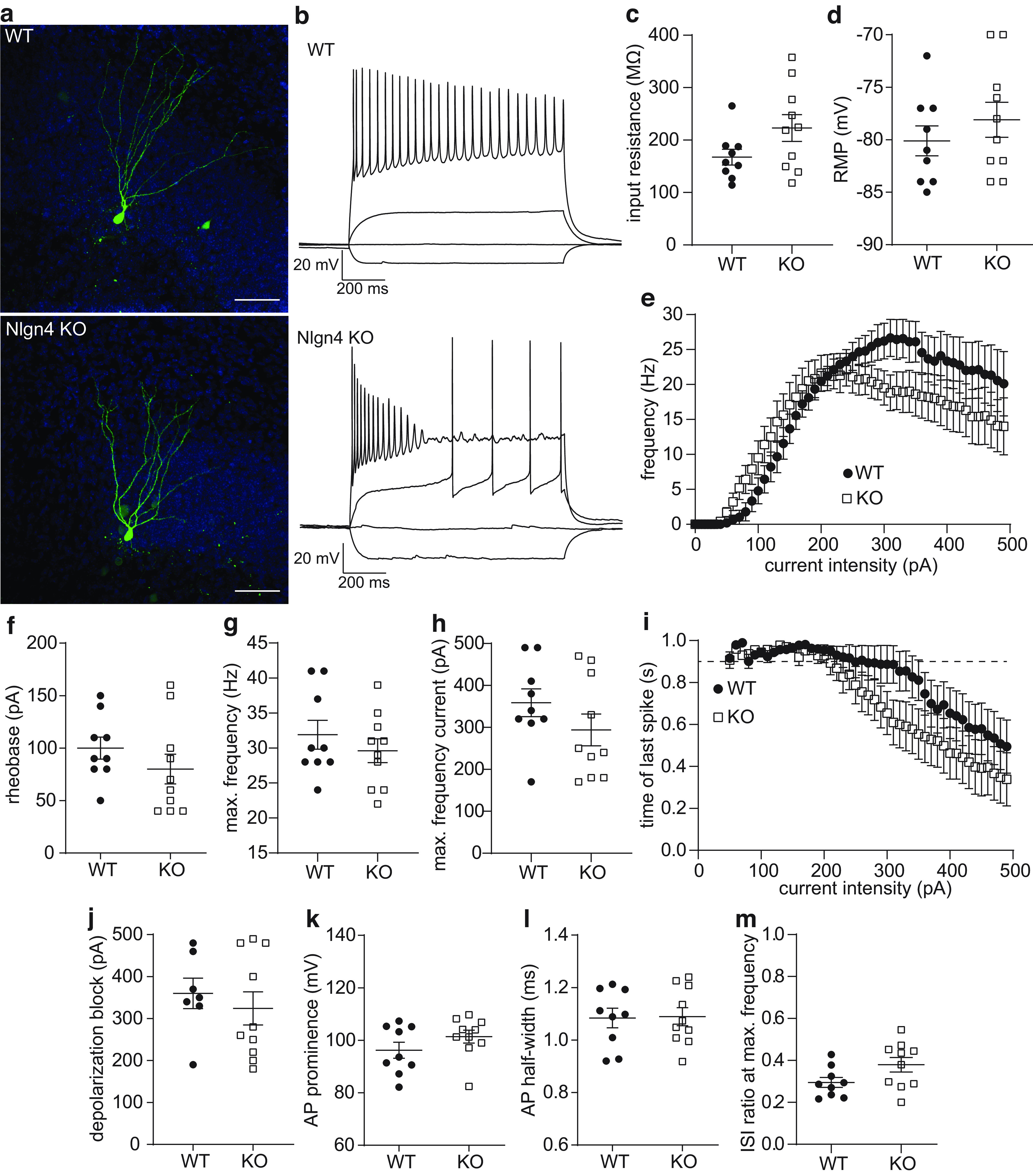
Unaltered passive electrotonic properties and subtle alterations in excitability in dentate granule cells from Nlgn4-deficient slice cultures. ***a***, *Post hoc* identified recorded granule cells from a WT and a Nlgn4-deficient slice culture (maximum intensity *z*-projection). Scale bar, 50 μm. ***b***, Voltage traces from one WT and one KO granule cell during −50, 0, 50, and 300 pA current injections. Note that the Nlgn4-deficient granule cell commenced spiking and entered depolarization block at lower current intensities compared with the WT cell. ***c***, ***d***, The input resistance (unpaired *t* test with Welch’s correction, *p *=* *0.082; ***c***) and the resting membrane potential (unpaired *t* test with Welch’s correction, *p *=* *0.37; ***d***) did not differ significantly between WT (*n *=* *9 cells from 7 slice cultures) and KO (*n *=* *10 cells from 8 slice cultures) granule cells. ***e***, The spike frequency plotted against the current intensity (*F–I* curve) reveals a significant interaction effect (WT granule cells, *n *=* *9; KO granule cells, *n* = 10; mixed-effects model, *p *=* *0.0031), but no effect of genotype (*p *=* *0.28) on firing frequency. ***f–h***, Comparison of the rheobase (unpaired *t* test with Welch’s correction, *p *=* *0.27; ***f***), the maximum firing frequency (unpaired *t* test with Welch’s correction, *p *=* *0.40; ***g***), and the current at which the maximum firing frequency was attained (unpaired *t* test with Welch’s correction, *p *=* *0.21; ***h***) reveal no significant differences between WT granule cells (*n *=* *9) and KO granule cells (*n *=* *10). ***i***, The time of the last spike during the 1 s current injection as a function of the current intensity showed that WT granule cells (*n *=* *9) appeared to enter depolarization block (represented by the dotted line at 0.9 s) at higher intensities than Nlgn4-deficient granule cells (*n *=* *10). ***j***, The mean current intensity during which cells entered depolarization block was not significantly different (WT granule cells, *n *=* *7; KO granule cells, *n* = 10; Mann–Whitney test, *p *=* *0.67). ***k***, ***l***, Neither the action potential prominence (Mann–Whitney test, *p *=* *0.28; ***k***) or the width at half-maximal prominence (unpaired *t* test with Welch’s correction, *p *=* *0.92; ***l***) differed significantly between WT granule cells (*n *=* *9) and KO granule cells (*n *=* *10). ***m***, The ISI ratio at the maximum firing frequency also revealed no significant difference between WT granule cells (*n *=* *9) and KO granule cells (*n *=* *10; unpaired *t* test with Welch’s correction, *p *=* *0.062).

To better interpret our *in vivo* findings regarding the increased network inhibition in the dentate gyrus, we performed patch-clamp recordings of individual granule cells in organotypic entorhino-hippocampal slice cultures prepared from WT and Nlgn4 KO mice (Fig. 5*a*). We examined the passive and active electrotonic properties of single granule cells using current-clamp recordings of the membrane voltage in combination with somatic current injections of increasing intensity (Fig. 5*b*). In these experiments, we used a potassium gluconate-based intracellular solution without calcium buffers to avoid influencing the frequency of action potential discharge (Madison and Nicoll, 1984).

The input resistance, as determined by the linear fit of the voltage differences during hyperpolarizing current injections, was higher in Nlgn4 KO granule cells compared with WT cells [Fig. 5*c*; KO (223.0 ± 25.6 MΩ; 95% CI, 165.1, 280.9) vs WT (167.5 ± 14.9 MΩ; 95% CI, 133.1, 201.9)]^n^, potentially leading to a higher excitability of these neurons, but this difference did not reach statistical significance (unpaired *t* test with Welch’s correction, *p *=* *0.082). The resting membrane potential was similar in Nlgn4 KO (−78.1 ± 1.7 mV; 95% CI, −74.3, −81.9; *n *=* *10 cells from eight slice cultures) and WT (−80.1 ± 1.4 mV; 95% CI, −76.8, −83.4; *n *=* *9 cells from seven slice cultures; unpaired *t* test with Welch’s correction, *p *=* *0.37) granule cells (Fig. 5*d*)^o^. Based on these findings, we conclude that the passive electrotonic properties of granule cells are not strongly affected by the knockout of Nlgn4.

Next, we examined the *F–I* relationship in individual granule cells by measuring the number of action potentials fired during depolarizing current injections (Fig. 5*e*). Analyzing the resulting *F–I* curves with a mixed-effects model revealed a significant interaction effect of the current intensity and the genotype (*p *=* *0.0031), meaning that the difference between Nlgn4-deficient and WT granule cells was dependent on the current intensity. However, the overall effect of the genotype was not significant (*p *=* *0.28). We noticed that the Nlgn4-deficient granule cells appeared to start firing action potentials at a lower current intensity, so we compared the current intensity at which the first action potential was elicited (rheobase). The rheobase was slightly lower in Nlgn4-deficient granule cells (Fig. 5*f*; 80.0 ± 14.1 vs 100.0 ± 10.4 pA for WT), but the 95% CIs overlapped considerably [KO (95% CI, 48.0, 112.0) vs WT (95% CI, 76.0, 124.0)], and the *t* test indicated no significant changes (*p *=* *0.27)^p^. At higher current intensities, the WT granule cells exhibited a higher firing frequency, but neither the maximum firing frequency [Fig. 5*g*; WT (31.9 ± 2.1 Hz; 95% CI, 27.1, 36.7) vs KO (29.6 ± 1.7 Hz; 95% CI, 25.8, 33.4; unpaired *t* test with Welch’s correction, *p *=* *0.40)]^q^ nor the current intensity to reach the maximum firing frequency [Fig. 5*h*; KO (294.0 ± 37.8 pA; 95% CI, 208.5, 379.5) vs WT (358.9 ± 33.1; 95% CI, 282.6, 435.2); unpaired *t* test with Welch’s correction, *p *= 0.21]^r^ differed significantly between genotypes.

Since the reduced firing frequencies in the KO group could be caused by the cells entering depolarization block at a lower current intensity, we also examined the time the last action potential was fired as a function of the current intensity (Fig. 5*i*). The current at which the cell entered depolarization block also revealed no significant differences between genotypes [Fig. 5*j*; KO (324.0 ± 40.0 pA; 95% CI, 234.6, 413.4) vs WT (360.0 ± 36.1 pA; 95% CI, 271.6, 448.4; Mann–Whitney test, *p *=* *0.67)]^s^. However, it should be noted that all Nlgn4-deficient granule cells entered depolarization block eventually, while two WT granule cells fired continuously without entering depolarization block during any of the tested current intensities. Therefore, we also analyzed the probability of entering depolarization block in the range of stimulation intensities tested (χ^2^ test of independence, χ^2^ = 2.5, *p *=* *0.12; Table 1). Together, these results indicate the Nlgn4-deficient granule cells trend in a more excitable direction, which could be related to their slightly but not significantly higher input resistance, but also tend to enter depolarization block at lower stimulation intensities compared with WT granule cells.

**Table 1:** χ^2^ Contingency table for entry into depolarization block, df = 1

	Entered depolarization block?		Total
	Yes	No	
WT	7	2	9
KO	10	0	10
Total	17	2	19

Action potentials are initiated at the axon initial segment, a specialized region containing a wide variety of ion channels that regulate different aspects of action potential firing. For instance, the action potential amplitude is dependent on the density of voltage-gated sodium channels in the axon initial segment, whereas the action potential half-width is regulated by K_v_1 voltage-gated potassium channels, and K_v_7 potassium channels impact spike frequency adaptation (Kole and Stuart, 2012). We therefore compared the properties of individual action potentials at the rheobase current to detect possible defects in action potential generation in the Nlgn4-deficient granule cells. The action potential prominence was slightly, but not significantly, higher in the KO (Fig. 5*k*, prominence; KO: 101.4 ± 2.5 mV; 95% CI, 95.8, 107.0; WT: 96.2 ± 3.1 mV; 95% CI, 89.2, 103.3; Mann–Whitney test, *p *=* *0.28)^t^. The action potential width at half-prominence was extremely similar (Fig. 5*l*; KO: 1.09 ± 0.03 ms; 95% CI, 1.01, 1.17; WT: 1.08 ± 0.04 ms; 95% CI, 1.00, 1.17; unpaired *t* test with Welch’s correction, *p *=* *0.92)^u^. Based on these two results, we concluded that the generation of action potentials was not affected by the deletion of Nlgn4. Both Nlgn4-deficient and WT granule cells exhibited spike frequency adaptation, which was quantified by the ratio of the first ISI to the last ISI. The results of the mixed model were inconclusive because the quantile–quantile plots revealed a strong deviation from normality. We therefore chose to compare the ISI ratios at the maximum firing frequency (Fig. 5*m*). This value was slightly higher in Nlgn4-deficient granule cells (0.38 ± 0.03; 95% CI, 0.30, 0.46) compared with WT granule cells (0.29 ± 0.02; 95% CI, 0.24, 0.35), and approached significance (unpaired *t* test with Welch’s correction, *p *=* *0.062)^v^.

### Excitatory and inhibitory synaptic transmission is unaltered in Nlgn4-deficient dentate granule cells

**Figure 6.**
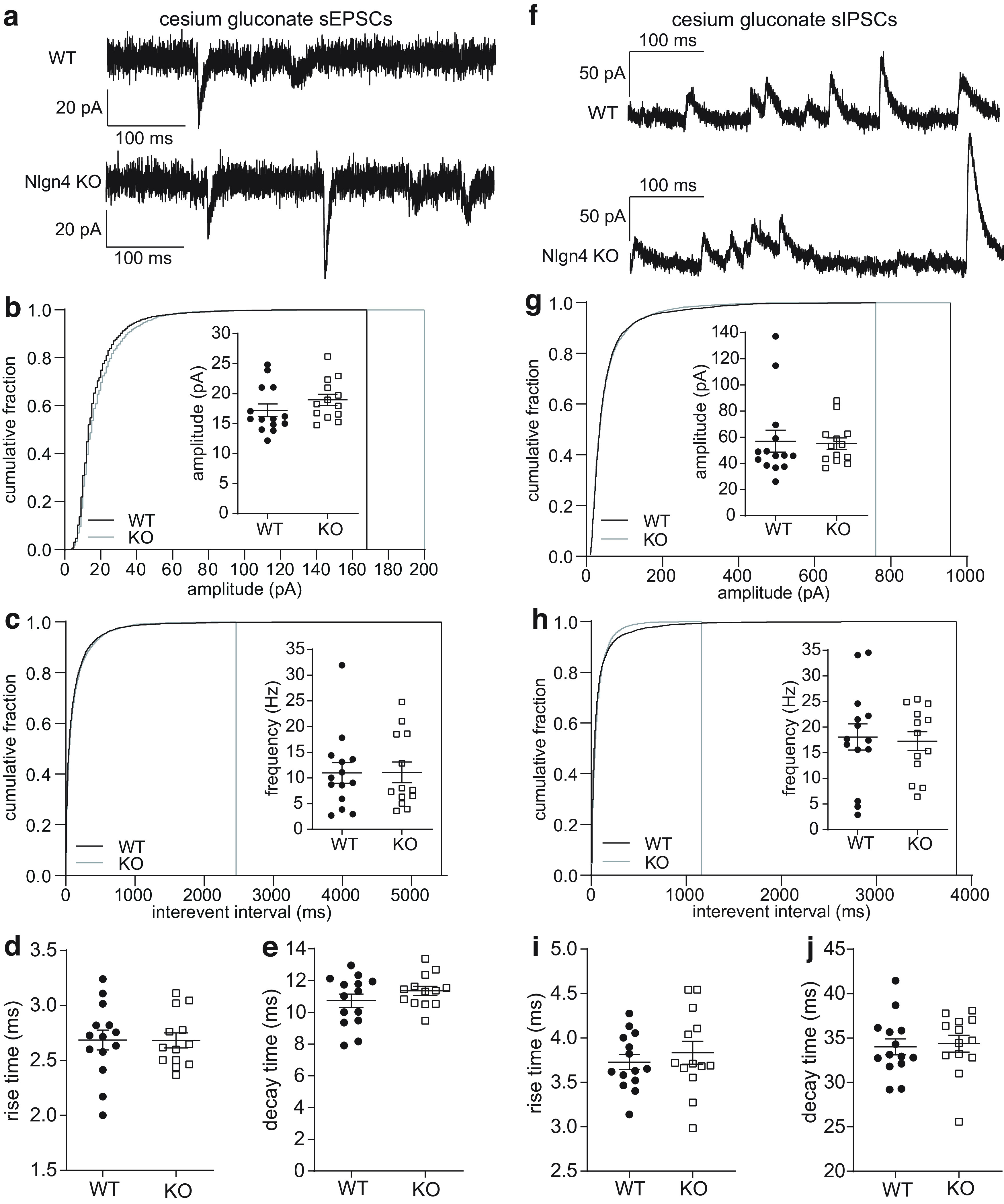
Comparable excitatory and inhibitory synaptic transmission in dentate granule cells from Nlgn4-deficient and wild-type slice cultures. ***a***, Representative sEPSC traces from one WT and one Nlgn4-deficient granule cell recorded using a cesium gluconate-based intracellular solution at a holding potential of −60 mV. ***b***, Cumulative plots and mean error plots reveal a similar distribution and mean of the sEPSC amplitude in WT granule cells (*n *=* *14) and Nlgn4-deficient granule cells (*n *=* *13; Mann–Whitney test, *p *=* *0.14). ***c***, Cumulative plots reveal a similar distribution of the sEPSC interevent interval, and mean error plots show a similar mean sEPSC frequency in WT granule cells (*n *=* *14) and Nlgn4-deficient granule cells (*n *=* *13; Mann–Whitney test, *p *=* *0.94). ***d***, ***e***, Comparison of sEPSC rise times (unpaired *t* test with Welch’s correction, *p *=* *0.97; ***d***) and sEPSC decay times (unpaired *t* test with Welch’s correction, *p *=* *0.23; ***e***) in WT granule cells (*n *=* *14) and Nlgn4-deficient granule cells (*n *=* *13) revealed no significant differences. ***f***, Representative granule cell sIPSC traces from organotypic slice cultures prepared from WT and Nlgn4 KO mice recorded with a cesium gluconate-based intracellular solution at a holding potential of 10 mV. ***g***, Cumulative plots and mean error plots reveal a similar distribution and mean of the sIPSC amplitudes in WT granule cells (*n *=* *14) and Nlgn4-deficient granule cells (*n *=* *13; Mann–Whitney test, *p *=* *0.58). ***h***, Cumulative plots reveal a similar distribution of the interevent intervals, and mean error plots show a similar sIPSC frequency in WT granule cells (*n *=* *14) and Nlgn4-deficient granule cells (*n *=* *13; unpaired *t* test with Welch’s correction, *p *=* *0.80). ***i***, ***j***, There were no significant differences in the rise time (unpaired *t* test with Welch’s correction, *p *=* *0.50; ***i***) or decay time (Mann–Whitney test, *p *=* *0.33; ***j***) between WT granule cells (*n *=* *14) and Nlgn4-deficient granule cells (*n *=* *13).

To corroborate our *in vivo* findings of unaltered excitatory synaptic transmission in the dentate gyrus, we measured spontaneous EPSCs from granule cells in organotypic entorhino-hippocampal slice cultures (Fig. 6*a*). While the reduction in leak conductance by the intracellular diffusion of cesium is unable to fully compensate for the space-clamp problem of somatic voltage clamp, it improves the voltage control over distal dendrites (Williams and Mitchell, 2008). Therefore, we used a cesium gluconate-based intracellular solution in these experiments to more accurately measure synaptic events.

We recorded sEPSCs at a holding potential of −60 mV in the presence of d-APV to isolate the AMPAR currents. There was no significant difference in the amplitude (Fig. 6*b*; WT: 17.22 ± 1.04 pA; 95% CI, 14.97, 19.47; *n *=* *14 cells from 14 slice cultures; KO: 18.97 ± 0.94 pA; 95% CI, 16.93, 21.01; *n *=* *13 cells from 13 slice cultures; unpaired *t* test with Welch’s correction, *p *=* *0.22)^w^ or frequency (Fig. 6*c*; WT: 10.99 ± 2.01 Hz; 95% CI, 6.65, 15.32; KO: 11.09 ± 2.00 Hz; 95% CI, 6.73, 15.44; Mann–Whitney test, *p *= 0.94)^x^ of the sEPSCs, and we also found no difference in the rise time (Fig. 6*d*; WT: 2.69 ± 0.09 ms; 95% CI, 2.49, 2.88; KO: 2.68 ± 0.07 ms; 95% CI, 2.53, 2.83; unpaired *t* test with Welch’s correction, *p *=* *0.97)^y^ or decay time (Fig. 6*e*; WT: 10.73 ± 0.42 ms; 95% CI, 9.81, 11.64; KO: 11.36 ± 0.29 ms; 95% CI, 10.73, 11.98; unpaired *t* test with Welch’s correction, *p *=* *0.23)^z^.

In addition to a decrease in the granule cell excitability, the difference in paired-pulse inhibition we observed *in vivo* could be caused by an increase in inhibitory synaptic strength in Nlgn4-deficient mice. To test this hypothesis, we recorded sIPSCs from the same granule cells we used for the sEPSC recordings at a holding potential of 10 mV (Fig. 6*f*). The distribution of the sIPSC amplitudes and interevent intervals was similar in WT and Nlgn4-deficient granule cells (Fig. 6*g*,*h*). We found no significant difference in the mean amplitude (Fig. 6*g*; WT: 56.97 ± 8.35 pA; 95% CI, 38.93, 75.00; *n *=* *14; KO: 55.05 ± 4.44 pA; 95% CI, 45.37, 64.73; *n *=* *13; Mann–Whitney test, *p *=* *0.58)^aa^, frequency (Fig. 6*h*; WT: 18.07 ± 2.56 Hz; 95% CI, 12.54, 23.59; KO: 17.24 ± 1.88 Hz; 95% CI, 13.15, 21.32; unpaired *t* test with Welch’s correction, *p *=* *0.80)^bb^, rise time (Fig. 6*i*; WT: 3.73 ± 0.08 ms; 95% CI, 3.55, 3.91; KO: 3.83 ± 0.13 ms; 95% CI, 3.55, 4.12; unpaired *t* test with Welch’s correction, *p *=* *0.50)^cc^, or decay time (Fig. 6*j*; WT: 34.00 ± 0.90 ms; 95% CI, 32.06, 35.94; KO: 34.37 ± 0.95 ms; 95% CI, 32.31, 36.43; Mann–Whitney test, *p *=* *0.33)^dd^ of the sIPSCs. Based on these results, we concluded that both excitatory and inhibitory transmission in granule cells is not affected by the loss of Nlgn4 in organotypic slice cultures.

### Excitation–inhibition balance is preserved in Nlgn4-deficient dentate granule cells

**Figure 7.**
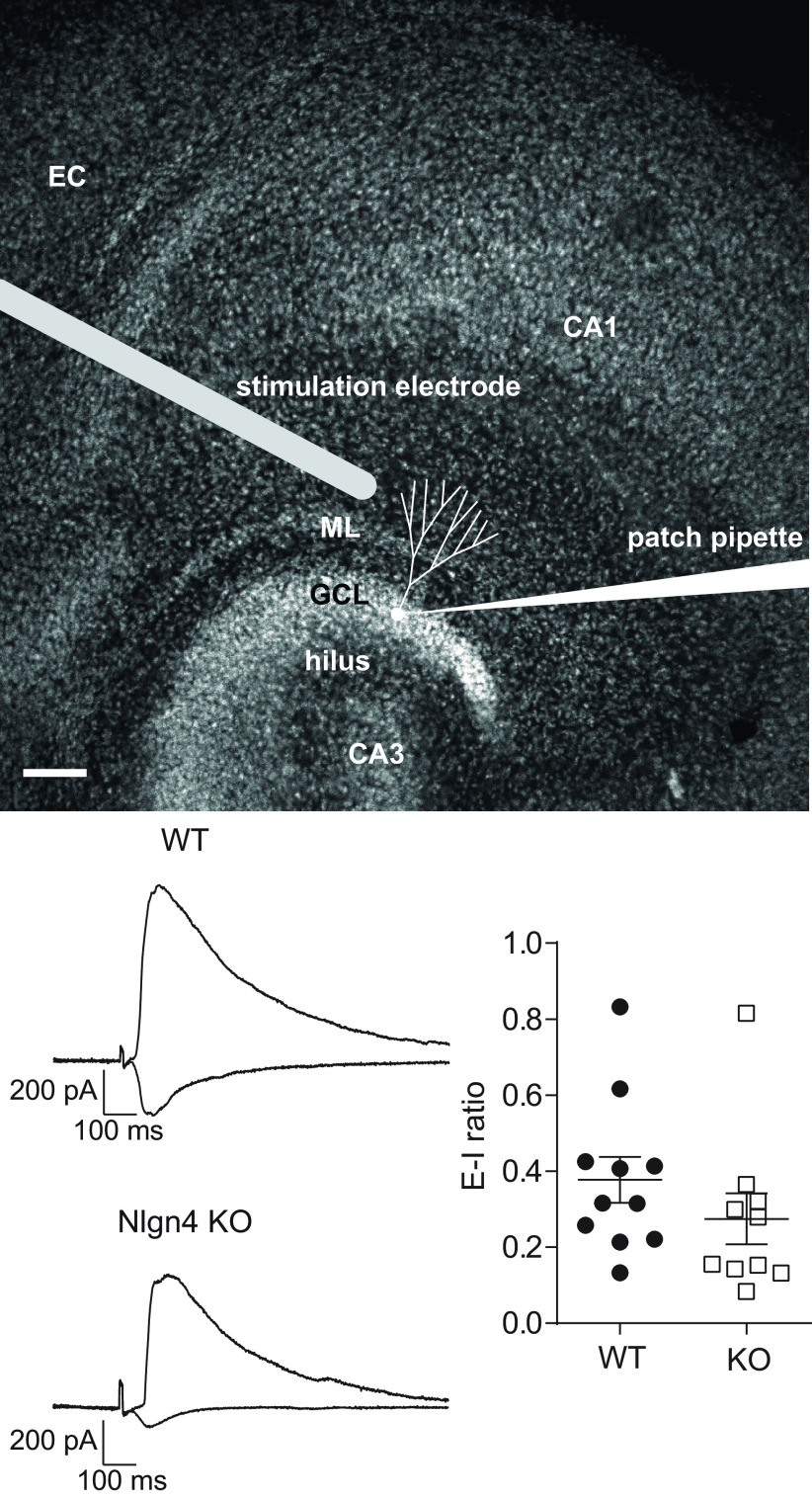
Excitation–inhibition balance is preserved in Nlgn4-deficient dentate granule cells. Top, Relative location of the stimulation electrode in the molecular layer of an organotypic entorhino-hippocampal slice culture. EC, Entorhinal cortex; GCL, granule cell layer; ML, molecular layer. Scale bar, 100 μm. Bottom left, Representative EPSCs and IPSCs evoked by molecular layer stimulation recorded in the same granule cell in slice cultures prepared from WT and Nlgn4 KO mice. Bottom right, The excitation/inhibition ratios did not differ significantly between WT granule cells (*n *=* *11) and Nlgn4-deficient granule cells (*n *=* *10; Mann–Whitney test, *p *=* *0.13).

Previous studies reported a reduced cortical E/I ratio in Nlgn4 KO mice (Delattre et al., 2013; Unichenko et al., 2018), which fits with the increased network inhibition we observed *in vivo*; however, these studies had not determined the E/I ratio of individual neurons. Using the cesium gluconate-based intracellular solution, we adjusted the holding potential to record EPSCs (at −60 mV) or IPSCs (at 10 mV) in response to the same stimulation intensity and to compute the E/I ratio of individual granule cells. We observed a slightly lower mean E/I ratio in Nlgn4 KO granule cells (Fig. 7; 0.27 ± 0.07; 95% CI, 0.12, 0.43; *n *=* *10 cells from 10 slice cultures) compared with WT cells (0.38 ± 0.06; 95% CI, 0.24, 0.51; *n *=* *11 cells from 11 slice cultures), but this difference was not significant (Mann–Whitney test, *p *=* *0.13)^ee^.

### Homeostatic synaptic plasticity in dentate granule cells is not affected by the loss of Nlgn4

**Figure 8.**
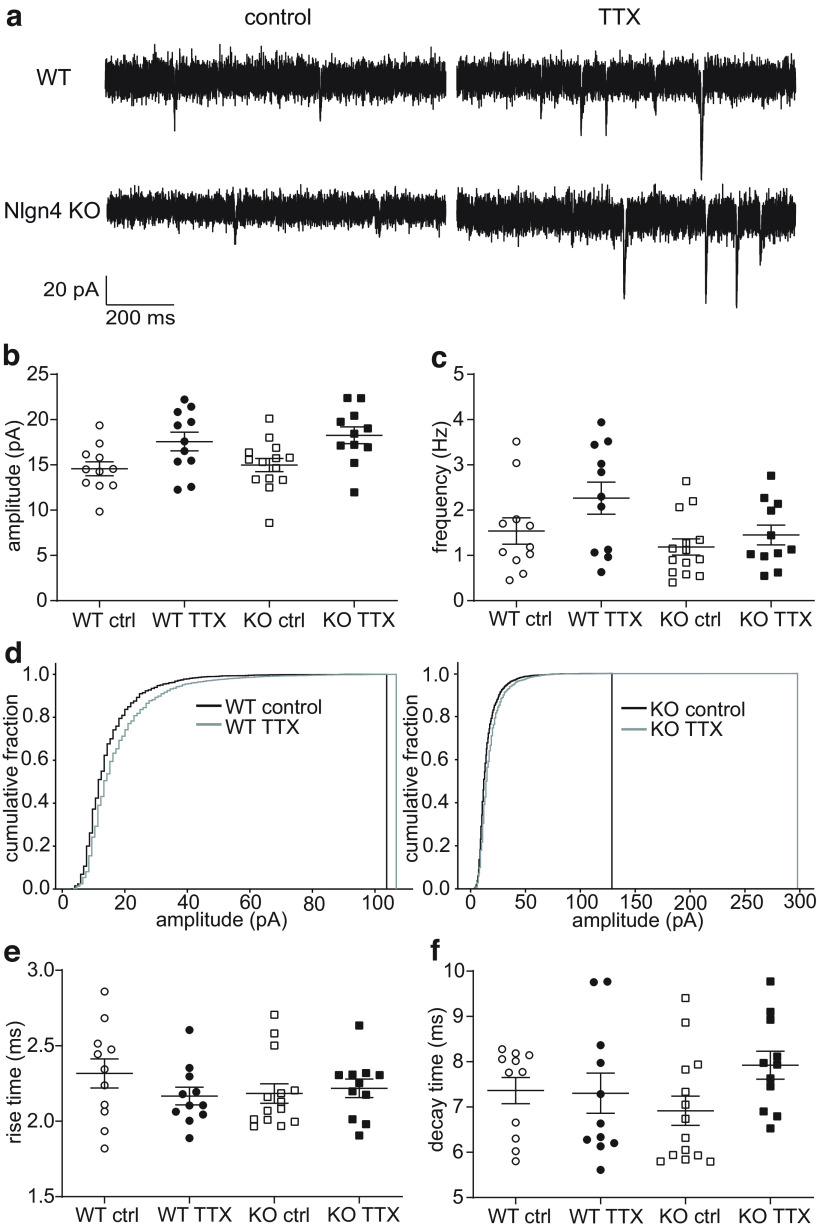
Homeostatic synaptic plasticity in dentate granule cells is not affected by the loss of Nlgn4. ***a***, Representative granule cell mEPSC traces from control and TTX-treated slice cultures prepared from WT and Nlgn4 KO mice. ***b***, mEPSC amplitudes were increased in TTX-treated slice cultures compared with control slice cultures from both WT mice (*n *=* *11 cells from 10 control slice cultures; *n* = 11 cells from 10 TTX-treated slice cultures) and KO mice (*n *=* *14 cells from 11 control slice cultures and *n *=* *11 cells from 9 TTX-treated slice cultures; two-way ANOVA: *p *=* *0.0008 for treatment, *p *=* *0.53 for genotype, and *p *=* *0.88 for interaction). ***c***, The mEPSC frequency differed between genotypes, but not treatments (two-way ANOVA: *p *=* *0.066 for treatment, *p *=* *0.031 for genotype, *p *=* *0.39 for interaction). ***d***, Cumulative plots reveal a shift toward higher amplitudes in the TTX-treated slice cultures. ***e***, ***f***, Neither the mEPSC rise time (two-way ANOVA: *p *=* *0.42 for treatment, *p *=* *0.57 for genotype, *p *=* *0.20 for interaction; ***e***) nor the mEPSC decay time (two-way ANOVA: *p *=* *0.18 for treatment, *p *=* *0.81 for genotype, *p *=* *0.13 for interaction; ***f***) differed based on the treatment or the genotype.

Our *in vivo* data had shown that Hebbian synaptic plasticity was largely unaffected by the deletion of Nlgn4, but it was unclear whether this finding generalized to other forms of synaptic plasticity operating with different molecular mechanisms. Homeostatic synaptic plasticity is a compensatory mechanism to normalize synaptic activity in response to a reduction in synaptic input (Turrigiano, 2012). Interestingly, impairments of homeostatic plasticity have been reported in several ASD mouse models (Nelson and Valakh, 2015), so we hypothesized that Nlgn4 might be involved in synaptic upscaling. We induced homeostatic synaptic upscaling by blocking synaptic activity with 2 μm TTX over a period of 2 d. Control slice cultures were treated with the same volume of water for the same duration of time. mEPSCs were recorded with a potassium gluconate-based intracellular solution in the presence of TTX, d-APV, and gabazine (Fig. 8*a*). The activity blockade led to an increase in mEPSC amplitude compared with controls for both WT (control: 14.58 ± 0.77 pA; 95% CI, 12.84, 16.31; *n *=* *11 cells from 10 slice cultures; TTX: 17.58 ± 1.04 pA; 95% CI, 15.27, 19.89; 11 cells from 10 slice cultures) and KO (control: 14.99 ± 0.73 pA; 95% CI, 13.41, 16.57; 14 cells from 11 slice cultures; TTX: 18.26 ± 0.93 pA; 95% CI, 16.20, 20.33; 11 cells from 9 slice cultures) granule cells (Fig. 8*b*)^ff^. The treatment accounted for most of the difference in amplitudes (two-way ANOVA, *p *=* *0.0008), while the genotype (*p *=* *0.53) and interaction (*p *=* *0.88) effects were not significant. This increase in the mean amplitude was also reflected in the cumulative probability plots, which showed a rightward shift in the TTX group. The mEPSC frequency was lower in the KO slice cultures in both the control (WT: 1.54 ± 0.29 Hz; 95% CI, 0.89, 2.19; KO: 1.19 ± 0.18 Hz; 95% CI, 0.80, 1.58) and TTX (WT: 2.27 ± 0.35 Hz; 95% CI, 1.48, 3.06; KO: 1.45 ± 0.22 Hz; 95% CI, 0.96, 1.94) conditions (Fig. 8*d*). The genotype effect was significant (two-way ANOVA, *p *=* *0.031), but the Bonferroni-corrected post-tests revealed no significant differences between WT and Nlgn4 KO granule cells^gg^. The treatment effect approached significance (*p *=* *0.066), but the interaction effect was not significant (*p *=* *0.39). Neither the mEPSC rise time (Fig. 8*e*; two-way ANOVA with Bonferroni’s multiple-comparisons test; *p *= 0.42 for treatment, *p *=* *0.57 for genotype, *p *=* *0.20 for interaction)^hh^ nor the mEPSC decay time (Fig. 8*f*; two-way ANOVA with Bonferroni’s multiple-comparisons test; *p *=* *0.18 for treatment, *p *=* *0.81 for genotype, *p *=* *0.13 for interaction)^ii^ differed based on the treatment or the genotype. Therefore, we concluded that homeostatic synaptic plasticity is preserved in granule cells from Nlgn4-deficient slice cultures.

## Discussion

E–I imbalance is one of the most widely studied mechanistic explanations of ASD (Nelson and Valakh, 2015). We found that the Nlgn4 KO mouse, a construct-valid and face-valid autism model, exhibits a small but significant increase in network inhibition in the dentate gyrus, resulting in a decreased E/I ratio. Since the increased paired-pulse inhibition we observed could be caused by an increase in inhibitory synaptic input and/or a decrease of the intrinsic excitability in the dentate granule cells, we extended our approach to an in-depth analysis *in vitro* to differentiate between these possibilities. Interestingly, the granule cells in Nlgn4-deficient organotypic slice cultures showed a tendency toward greater, not lower, excitability and inhibitory synaptic transmission was unaltered. Therefore, our findings on the level of single granule cells in neonatal organotypic slice cultures cannot explain the differences in inhibition in the intact network *in vivo.* Instead, the increase in inhibition observed in the mature network *in vivo* could be related to a compensatory increase in the ratio of Nlgn2 to Nlgn1, as previously reported for hippocampal synaptosomes from adult Nlgn4 KO mice (Hammer et al., 2015) or to a difference in inhibitory interneuron function.

**Figure 9.**
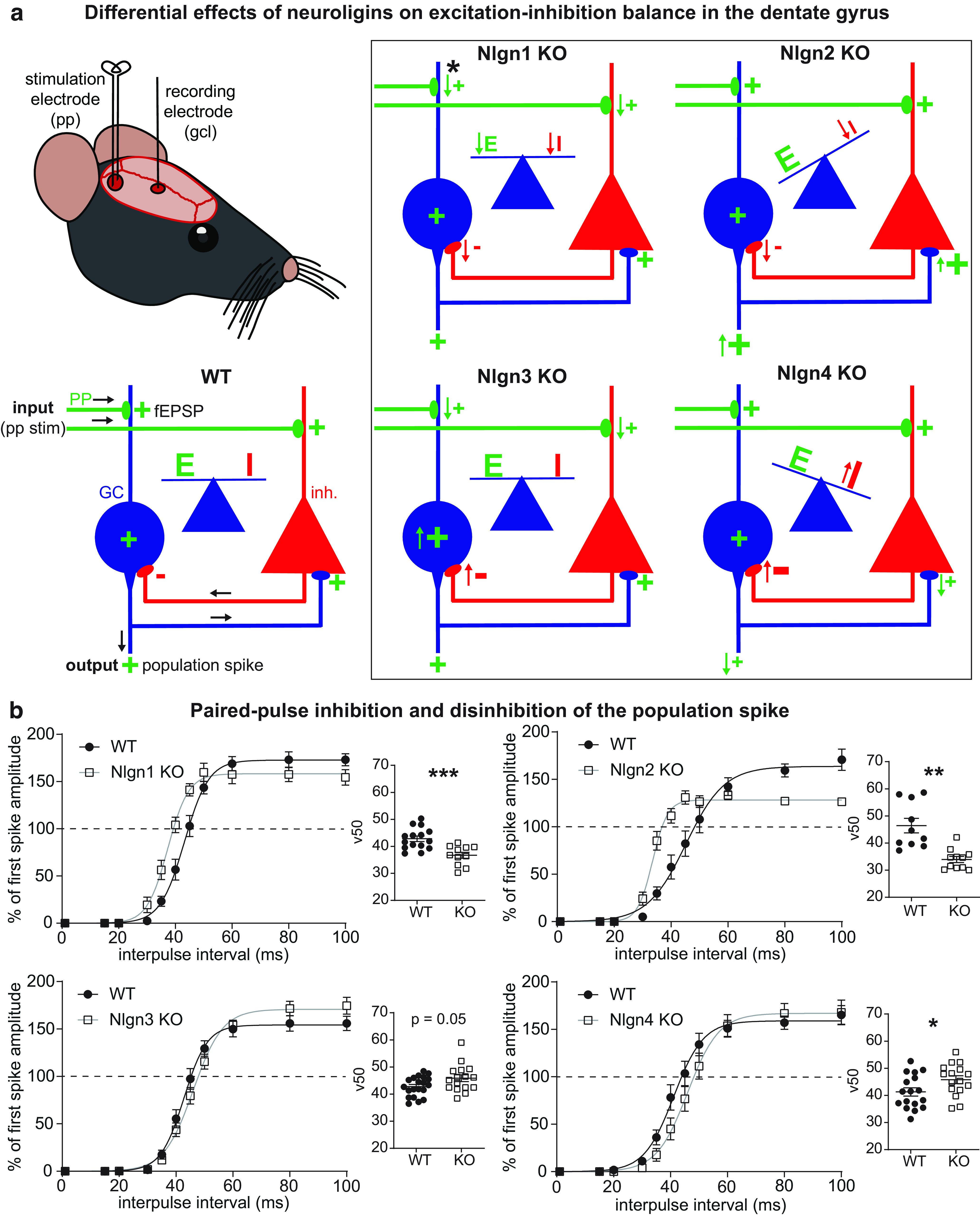
Neuroligins differentially affect E–I balance in the dentate gyrus. ***a***, *In vivo* recordings of evoked fEPSPs from the granule cell layer (gcl) can be used to assess excitation–inhibition balance in the dentate gyrus. In WT mice, E and I are carefully balanced. In Nlgn1 KO mice, both E and I are reduced, so the net effect on the granule cell population spike output is zero (Jedlicka et al., 2015). In Nlgn2 KO mice, excitation is unchanged, but inhibition is reduced, resulting in an increase in the granule cell excitability and an E–I imbalance (Jedlicka et al., 2011). In Nlgn3 KO mice, the reduced excitatory input and slightly increased inhibition is compensated by an increase in the intrinsic neuronal excitability of the granule cells, thereby preserving the E–I balance (Muellerleile et al., 2022). In Nlgn4 KO mice, excitatory inputs are unchanged, but network inhibition is increased, resulting in an E–I imbalance. *Of note, the only defect in the induction of long-term potentiation at perforant path–granule cell synapses was observed in Nlgn1 KO mice. GC, Granule cell; inh., inhibitory interneuron; PP, perforant path. ***b***, Paired-pulse inhibition of the granule cell population spike induced by double-pulse stimulation at the maximal stimulation intensity (800 μA) is reduced in Nlgn1 KO and Nlgn2 KO mice, but slightly enhanced in Nlgn3 KO mice and significantly enhanced in Nlgn4 KO mice compared with WT littermate controls. The shift in the curve relating the degree of inhibition of the second population spike to the interpulse interval was quantified by the v50 parameter of a Boltzmann fit (unpaired *t* test with Welch’s correction, *p < 0.05, **p < 0.01, ***p < 0.001).

Previous studies have shown that the four neuroligin proteins regulate different aspects of synaptic transmission and network excitability in mice. Remarkably, each of the four neuroligins (Nlgn1–4) exerts a characteristic effect on both intrinsic cellular and network activity in the dentate gyrus *in vivo* (Fig. 9*a*). The granule cell output, measured by the amplitude of the population spike, is determined by the complex interplay of the excitatory synaptic strength (quantified by the fEPSP slope), the intrinsic neuronal excitability of the granule cells, and feedback and feedforward inhibition from local interneurons (assessed with a paired-pulse protocol at maximal stimulation intensity; Fig. 9*b*)^jj^. Thus, the population spike can be used as a readout of the dentate E–I balance (Jedlicka et al., 2018). Nlgn1 KO mice exhibit a reduction in both the fEPSP slope and paired-pulse inhibition, but an unchanged population spike output, thus preserving the network E–I balance (Jedlicka et al., 2015). Synaptic strength measured by the fEPSP slope is unchanged in Nlgn2 KO mice, but paired-pulse inhibition is strongly decreased and the population spike output is strongly increased, leading to an increase in network excitation (Jedlicka et al., 2011). Nlgn3 KO mice also exhibit a reduction in the fEPSP slope, but show a tendency toward increased, rather than decreased, paired-pulse inhibition; indicating that the unchanged population spike output is the result of a compensatory increase in the intrinsic excitability (Muellerleile et al., 2022). In the present work, we show that Nlgn4 KO mice exhibit no differences in excitatory synaptic transmission, a tendency toward decreased population spike output and an increase in paired-pulse inhibition (Fig. 4) that was more pronounced than in the Nlgn3 KO, leading to an overall decrease in the E/I ratio in the dentate gyrus.

The mechanisms underlying the reduction in paired-pulse inhibition in the Nlgn1 and Nlgn2 KO mice are relatively straightforward to understand: the effect on paired-pulse inhibition can be explained by a reduction in excitatory synaptic strength onto inhibitory interneurons in Nlgn1 KO mice and a reduction in inhibitory synaptic strength in granule cells in Nlgn2 KO mice. In contrast, the increase in paired-pulse inhibition observed in the Nlgn3 and Nlgn4 KO mice is less clear mechanistically. In Nlgn3 KO mice, a disruption of tonic endocannabinoid signaling from granule cells to inhibitory interneurons might result in increased network inhibition, as has been demonstrated for the cholecystokinin (CCK)-expressing interneuron–cornu ammonis 1 (CA1) pyramidal cell synapse (Földy et al., 2013). Perhaps Nlgn4 also regulates endocannabinoid signaling to interneurons, but this would have to be tested using paired recordings of CCK interneurons and granule cells. Alternatively, Nlgn4 may regulate inhibitory synapses on parvalbumin (PV)-expressing interneurons, which perisomatically inhibit granule cells and thereby exert the greatest control over the granule cell excitability compared with other interneuron populations (Elgueta and Bartos, 2019). If Nlgn4 contributes to the assembly or maintenance of inhibitory synapses onto dentate interneurons, then the deletion of Nlgn4 might reduce the inhibition of interneurons, thereby enhancing downstream inhibition of granule cells. However, an effect of Nlgn4 on excitatory synaptic function in PV interneurons, as was shown for Nlgn3 in hippocampal area CA1 (Polepalli et al., 2017), also cannot be excluded based on the available evidence. Interestingly, while perisomatic inhibition and the recruitment of gephyrin and GABA_A_ receptors to inhibitory synapses in hippocampal area CA3 were reduced in Nlgn4 KO mice, hippocampal synaptosomal preparations revealed an increase in the Nlgn2/Nlgn1 ratio, possibly representing a compensatory response to the loss of Nlgn4 (Hammer et al., 2015). Since inhibitory synaptic function and, presumably, Nlgn2 expression was reduced in area CA3 of Nlgn4 KO mice, it stands to reason that other hippocampal subregions might exhibit an increase in Nlgn2 expression. Thus, the increased network inhibition we observed in the dentate gyrus could be related to a relative increase in the levels of Nlgn2, which would be expected to increase the strength of inhibitory synapses.

To our surprise, we did not observe any significant differences in inhibitory synaptic transmission (i.e., sIPSCs) in granule cells in organotypic slice cultures (Fig. 6). Both GABAergic (Delattre et al., 2013; Hammer et al., 2015; Unichenko et al., 2018) and glycinergic (Hoon et al., 2011; Zhang et al., 2018) transmission have been shown to be regulated by Nlgn4 in other brain regions, so our findings might reflect a regional differentiation of Nlgn4 function. However, the model systems and the ages of the animals also differed: we used organotypic slice cultures prepared from P5 mice, whereas the other studies used acute slices from young mice between P12 and P30. While we allowed the slice cultures to mature *in vitro* for 3 weeks before performing experiments, it is possible that the peak expression levels of Nlgn4 is not yet attained so difference between genotypes may be obscured in the slice cultures. Nlgn4 protein expression in the brain reaches a plateau 3 weeks after birth (Jamain et al., 2008), and Nlgn4 mRNA expression increases from P1 to P18 in the brainstem (Zhang et al., 2018). Thus, the increased paired-pulse inhibition in the dentate gyrus might result from altered network activity that occurs only at later developmental stages.

It was previously shown that increased paired-pulse inhibition in the dentate gyrus could also be caused by a reduction of voltage-gated sodium channels in the axon initial segment, thereby impairing the ability of the granule cells to generate action potentials (Winkels et al., 2009), but the *in vitro* experiments did not indicate any changes in the properties of individual action potentials. However, granule cells in Nlgn4-deficient cultures showed a tendency to enter depolarization block at lower current intensities compared with WT granule cells, but this difference was not significant (Fig. 5*i*, Table 1). Depolarization block is mediated by the interplay between the transient sodium and delayed-rectifier potassium currents (Bianchi et al., 2012) and may serve an important purpose in preventing network hyperexcitability. While somatic current injections do not represent normal physiological activity, currents >1 nA could be generated by the convergent input from only 3% of excitatory synapses on a CA1 pyramidal neuron (Bianchi et al., 2012), so current injections up to 500 pA are well within the physiological range. We also observed a slight, but not significant, increase in the interspike interval ratio of Nlgn4-deficient granule cells, reflecting a trend toward weaker spike frequency adaptation (Fig. 5*m*). Spike frequency adaptation is hypothesized to stabilize neuronal network dynamics (Barranca et al., 2019) and could play a role in the generation of gamma oscillations (Kilpatrick and Ermentrout, 2011). However, even if additional experiments with larger sample sizes were to confirm these trends as significant, they are unlikely to account for the observed increase in paired-pulse inhibition *in vivo* because of their small magnitude.

While some studies have demonstrated a role for Nlgn4 at excitatory synapses in mice (Delattre et al., 2013; Unichenko et al., 2018), we found no evidence of Nlgn4 involvement in excitatory synaptic transmission or plasticity *in vivo* or *in vitro*. We investigated presynaptic short-term plasticity with a paired-pulse protocol because a previous study had implicated the postsynaptic protein complex of Nlgn1 and PSD-95 in the regulation of presynaptic vesicle release probability at excitatory synapses in the rat hippocampus (Futai et al., 2007), and murine Nlgn4 also contains a PSD-95 binding site (Maxeiner et al., 2020). An increase in the level of paired-pulse facilitation at perforant path and granule cell synapses on basket cells, which could account for the increase in paired-pulse inhibition in the dentate gyrus (Thomas et al., 2005), cannot be excluded but appears unlikely given the lack of a significant effect on presynaptic plasticity at perforant path–granule cell synapses. We also detected no differences in the degree of LTP induction between KO and WT littermates (Fig. 2). The absence of an LTP deficit is consistent with the previously reported absence of deficits in hippocampal-dependent learning in Nlgn4 KO mice (Jamain et al., 2008). Furthermore, our analysis of the synaptic scaling experiments revealed no genotype differences in the amplitude of mEPSCs (Fig. 8), and the significant genotype effect on the mEPSC frequency might reflect differences in the variances rather than differences in the means (Table 2, nonsignificant *post hoc* comparisons). However, we cannot exclude a function for Nlgn4 at excitatory synapses since all these experiments used constitutive KO mice, which potentially experience confounding developmental or homeostatic compensation. Other studies of murine Nlgn4 found evidence for an involvement in excitatory synaptic transmission, but these results were obtained from cortical recordings (Delattre et al., 2013; Unichenko et al., 2018), and the synaptic localization of Nlgn4 might differ between the hippocampus and the cortex. The present results further underscore the differences between murine Nlgn4 and human NLGN4X, which was predominantly expressed at excitatory synapses in the brain regions and cell types studied thus far (Zhang et al., 2009; Chanda et al., 2016; Marro et al., 2019; Cast et al., 2021). While these differences could be specific to certain brain areas or cell types, differences in glycosylation could also be responsible for the differential synaptic localization because the murine Nlgn4 lacks an additional glycosylation site that is preserved in all human neuroligins (Cast et al., 2021). Nevertheless, even if the synaptic localization of Nlgn4 differs in human and murine neurons, common mechanisms might be involved in the generation of the behavioral symptoms observed in humans with ASD-related Nlgn4 mutations and Nlgn4 KO mice.

**Table 2:** Statistical table

	Data structure	Type of test	Difference between means or medians (WT − KO)		95% confidence interval of difference
a (PPF, % of first fEPSP)	Normal distribution	Bonferroni’s multiple-comparisons post-test	15 ms	−8.48	−33.95 to 17.00
			20 ms	−13.89	−36.03 to 8.25
			40 ms	−6.83	−21.29 to 7.64
			60 ms	−6.95	−21.89 to 7.98
			80 ms	−0.32	−10.48 to 9.84
			100 ms	−0.75	−14.63 to 13.13
b (IO slope, V/s)	Normal distribution	Bonferroni’s multiple-comparisons post-test	30 μA	0.06	−0.05 to 0.16
			50 μA	0.14	−0.15 to 0.43
			100 μA	0.19	−0.23 to 0.61
			150 μA	0.16	−0.32 to 0.63
			175 μA	0.14	−0.33 to 0.62
			200 μA	0.14	−0.31 to 0.59
			225 μA	0.09	−0.37 to 0.55
			250 μA	0.07	−0.42 to 0.56
			275 μA	0.09	−0.41 to 0.59
			300 μA	0.11	−0.33 to 0.55
			350 μA	0.12	−0.32 to 0.56
			400 μA	0.07	−0.38 to 0.53
			450 μA	0.05	−0.42 to 0.53
			500 μA	0.10	−0.39 to 0.58
			550 μA	0.04	−0.45 to 0.54
			600 μA	0.06	−0.42 to 0.55
			650 μA	0.04	−0.44 to 0.52
			700 μA	0.04	−0.46 to 0.54
			750 μA	0.08	−0.43 to 0.59
			800 μA	0.04	−0.42 to 0.50
c (LTP slope, % baseline)	Normal distribution	Welch’s *t* test (two-tailed)	0−10 min	3.57	−9.05 to 11.49
			50−60 min	1.22	−5.82 to 12.97
d (LTP spike, % baseline)	Normal distribution	Welch’s *t* test (two-tailed)	0−10 min	17.86	−35.18 to 28.50
			50−60 min	−3.34	−18.33 to 54.04
e (IO spike, mV)	Normal distribution	Bonferroni’s multiple-comparisons post-test	30 μA	0.00	
			50 μA	0.00	
			100 μA	0.42	−1.38 to 2.22
			150 μA	0.67	−1.13 to 2.46
			175 μA	0.64	−1.16 to 2.44
			200 μA	0.72	−1.07 to 2.52
			225 μA	1.13	−0.67 to 2.93
			250 μA	1.30	−0.50 to 3.10
			275 μA	0.77	−1.02 to 2.57
			300 μA	0.62	−1.18 to 2.41
			350 μA	0.39	−1.40 to 2.19
			400 μA	0.26	−1.53 to 2.06
			450 μA	0.70	−1.10 to 2.50
			500 μA	0.72	−1.08 to 2.52
			550 μA	0.83	−0.96 to 2.63
			600 μA	0.90	−0.90 to 2.69
			650 μA	0.64	−1.16 to 2.44
			700 μA	1.21	−0.59 to 3.00
			750 μA	0.77	−1.02 to 2.57
			800 μA	0.97	−0.82 to 2.77
f (spike onset, μA)	Normal distribution	Welch’s *t* test (two-tailed)	−41.54		−89.51 to 6.42
g (v50, V/s)	Normal distribution	Welch’s *t* test (two-tailed)	0.13		−0.18 to 0.43
h (PPI maximum, % of first spike amplitude)	Normal distribution	Bonferroni’s multiple-comparisons post-test	1 ms	0.00	
			15 ms	0.00	
			20 ms	−1.674	−5.249 to 1.902
			30 ms	−7.213	−19.24 to 4.814
			35 ms	−18.32	−49.92 to 13.28
			40 ms	−33.15	−86.87 to 20.58
			45 ms	−26.77	−82.97 to 29.43
			50 ms	−22.71	−78.16 to 32.75
			60 ms	2.72	−42.38 to 47.82
			80 ms	9.87	−29.05 to 48.79
			100 ms	2.61	−47.83 to 53.06
i (PPI maximum, interpolated IPI, ms)	Normal distribution	Welch’s *t* test (two-tailed)	50%	−4.03	−8.32 to 0.25
			75%	−4.18	−8.60 to 0.25
			100%	−4.22	−8.92 to 0.48
j (PPI maximum, v50, ms)	Normal distribution	Welch’s *t* test (two-tailed)	−4.52		−8.80 to −0.23
k (PPI min, % of first spike amplitude)	Normal distribution	Bonferroni’s multiple-comparisons post-test	1 ms	0.00	−26.62 to 68.60
			15 ms	0.00	−42.10 to 66.13
			20 ms	0.00	−87.42 to 113.8
			30 ms	20.99	−148.9 to 153.4
			35 ms	12.01	−188.3 to 168.2
			40 ms	13.21	−190.2 to 140.5
			45 ms	2.272	−268.3 to 276.5
			50 ms	−10.05	−166.4 to 135.9
			60 ms	−24.84	
			80 ms	4.10	
			100 ms	−15.25	
l (PPI minimum, interpolated IPI, ms)	Non-normal distribution	Mann–Whitney test (two-tailed)	50%	0.12	−8.83 to 6.94 (exact)
			75%	−0.26	−9.11 to 7.86 (exact)
			100%	0.05	−8.30 to 7.76 (exact)
m (PPI min, v50, ms)	Non-normal distribution	Mann–Whitney test (two-tailed)	−2.35		−7.61 to 4.39 (exact)
n (input resistance, MΩ)	Normal distribution	Welch’s *t* test (two-tailed)	−55.49		−118.9 to 7.96
o (resting membrane potential, mV)	Normal distribution	Welch’s *t* test (two-tailed)	−2.01		−6.63 to 2.60
p (rheobase, pA)	Normal distribution	Welch’s *t* test (two-tailed)	20.00		−17.21 to 57.21
q (maximum firing frequency, Hz)	Normal distribution	Welch’s *t* test (two-tailed)	2.29		−3.37 to 7.94
r (current at maximum firing frequency, pA)	Normal distribution	Welch’s *t* test (two-tailed)	64.89		−41.17 to 170.9
s (current at depolarization block, pA)	Unknown	Mann–Whitney test (two-tailed)	80.00		−110.0 to 170.0 (exact)
t (action potential prominence, mV)	Non-normal distribution	Mann–Whitney test (two-tailed)	−9.03		−14.98 to 3.91 (exact)
u (action potential half-width, ms)	Normal distribution	Welch’s *t* test (two-tailed)	−0.005		−0.11 to 0.10
v (ISI ratio at maximum firing frequency)	Normal distribution	Welch’s *t* test (two-tailed)	−0.08		−0.17 to 0.004
w (sEPSC amplitude, pA)	Normal distribution	Welch’s *t* test (two-tailed)	−1.75		−4.64 to 1.13
x (sEPSC frequency, Hz)	Non-normal distribution	Mann–Whitney test (two-tailed)	1.89		−5.04 to 5.14 (exact)
y (sEPSC rise time, ms)	Normal distribution	Welch’s *t* test (two-tailed)	0.004		−0.23 to 0.24
z (sEPSC decay time, ms)	Normal distribution	Welch’s *t* test (two-tailed)	−0.63		−1.69 to 0.43
aa (sIPSC amplitude, pA)	Non-normal distribution	Mann–Whitney test (two-tailed)	−7.32		−16.03 to 6.95 (exact)
bb (sIPSC frequency, Hz)	Normal distribution	Welch’s *t* test (two-tailed)	0.83		−5.72 to 7.39
cc (sIPSC rise time, ms)	Normal distribution	Welch’s *t* test (two-tailed)	−0.11		−0.43 to 0.21
dd (sIPSC decay time, ms)	Non-normal distribution	Mann–Whitney test (two-tailed)	−1.71		−3.60 to 1.79 (exact)
ee (E/I ratio)	Non-normal distribution	Mann–Whitney test (two-tailed)	0.10		−0.04 to 0.25 (exact)
ff (mEPSC amplitude, pA)	Normal distribution	Bonferroni’s multiple-comparisons post-test	Control	−0.42	−3.19 to 2.35
			TTX	−0.68	−3.61 to 2.25
gg (mEPSC frequency, Hz)	Normal distribution	Bonferroni’s multiple-comparisons post-test	Control	0.35	−0.48 to 1.19
			TTX	0.81	−0.07 to 1.70
hh (mEPSC rise time, ms)	Normal distribution	Bonferroni’s multiple-comparisons post-test	Control	0.13	−0.10 to 0.36
			TTX	−0.05	−0.29 to 0.19
ii (mEPSC decay time, ms)	Normal distribution	Bonferroni’s multiple-comparisons post-test	Control	0.44	−0.66 to 1.55
			TTX	−0.62	−1.79 to 0.55
jj (PPI maximum, v50, ms)	Normal distribution	Welch’s *t* test (two-tailed)	Nlgn1	6.118	3.001−9.235
			Nlgn2	12.49	6.145−18.83
			Nlgn3	−3.007	−6.145 to 0.0005
			Nlgn4	−4.515	−8.800 to −0.2307

PPI, paired-pulse inhibition; IO, input-output; IPI, interpulse interval.

Overall, the differences we observed in the Nlgn4 KO mice compared with WT littermates were very small relative to the differences observed in other neuroligin KOs, possibly because of compensation by other neuroligins. However, while immunostainings revealed an upregulation of Nlgn4 in the retina of Nlgn2 KO mice, suggesting that Nlgn4 is able to replace Nlgn2 at some inhibitory synapses, the expression of Nlgn2 was similar in WT and Nlgn4 KO retinae (Hoon et al., 2011). Furthermore, despite the increased Nlgn2/Nlgn1 ratio, inhibitory synaptic transmission was decreased in CA3 pyramidal cells from Nlgn4 KO mice (Hammer et al., 2015), which seems to preclude a complete compensation of Nlgn4 function by Nlgn2. While there are important differences regarding Nlgn4 expression in the hippocampus and the retina—Nlgn4 is predominantly localized to glycinergic postsynapses in the retina (Hoon et al., 2011), but it is primarily associated with a subset of GABAergic postsynapses in hippocampal area CA3 (Hammer et al., 2015)—it is noteworthy that Nlgn2 was unable to compensate for the loss of Nlgn4 in two different brain regions. Even if Nlgn2 could take over the function of Nlgn4, it is possible that the compensation may be misdirected such that the relative strength of inhibitory synaptic transmission is increased in the dentate gyrus, but not in area CA3, as discussed above.

Another explanation for the weaker effect of the Nlgn4 deletion on synaptic function in the dentate gyrus is the overall weaker expression of Nlgn4 protein compared with the other neuroligins. Nlgn4 makes up only 3% of the total neuroligin protein in the adult mouse brain (Varoqueaux et al., 2006), so any differences in the Nlgn4 KO are expected to be weaker compared with other neuroligin KOs. Furthermore, it was previously shown that Nlgn1 protein expression was downregulated in hippocampal synaptosomes from Nlgn3 KO mice, which could help explain the prominent defects in excitatory synaptic transmission observed in these mice (Muellerleile et al., 2022). Since Nlgn4 does not form heterodimers with other neuroligins (Poulopoulos et al., 2012), its deletion is unlikely to directly affect the expression of other neuroligins and thus have a narrower impact compared with the deletion of neuroligins that form heterodimers. Nevertheless, even small changes in the E–I balance of individual neurons can have profound effects on the network activity, as demonstrated by the decrease in gamma oscillations observed in the hippocampal subregion CA3 of Nlgn4-deficient mice (Hammer et al., 2015). The increased paired-pulse inhibition that was observed at interpulse intervals between 30 and 60 ms in the Nlgn4 KO mice would be expected to attenuate higher-frequency signals arriving from the entorhinal cortex, thus increasing the filtering capability of the dentate gyrus and possibly aiding in pattern separation (Thomas et al., 2005).

In conclusion, we provide evidence for an increased level of network inhibition in the dentate gyrus of adult Nlgn4 KO mice *in vivo* without any accompanying changes in the intrinsic excitability of the dentate granule cells, although whole-cell patch-clamp recordings in organotypic entorhino-hippocampal slice cultures revealed a trend toward decreased spike frequency adaptation and increased depolarization block. Intriguingly, the deletion of the fellow autism candidate gene Nlgn3 also leads to a slight increase in network inhibition in the dentate gyrus (Muellerleile et al., 2022), which suggests that increased inhibition might underlie some of the behavioral symptoms observed in Nlgn3 and Nlgn4 KO mice and in individuals with autism-associated neuroligin mutations.

## Synthesis

Reviewing Editor: Viji Santhakumar, University of California Riverside Decisions are customarily a result of the Reviewing Editor and the peer reviewers coming together and discussing their recommendations until a consensus is reached. When revisions are invited, a fact-based synthesis statement explaining their decision and outlining what is needed to prepare a revision will be listed below. The following reviewer(s) agreed to reveal their identity: Megan Wyeth.

Synthesis

The authors investigate the role of Nlgn4 on granule cells both in vivo and in slice cultures and identify little change apart from a modest increase in network inhibition during paired pulse stimulation at maximum intensity in vivo. Overall, the experiments are extensive, carefully conducted and complement the groups prior studies in Nlgn1-3 knockout mice and makes for a valuable comparison. While several parameters are examined both in vivo and in vitro were not significantly different between WT and KO, there were certain trends which appear interesting. However, the description tends to be confusing as conclusions about differences are suggested based on trends lacking statistical significance. Additionally, the evaluation of whole cell physiology in organotypic slices rather than acute slices from adult animals makes the findings in vitro and in vivo difficult to compare. Despite the focus on Nlgn4 in dentate, it is unclear whether Nlgn4 is expressed in dentate granule cells and possibly interneurons.

Major Comments

1. Is Nlgn4 even expressed by dentate gyrus granule cells? If not, this might not be the best cell type to be investigating. Evidence of Nlgn4 expression in dentate granule cells and/or interneurons would be important to demonstrate. Additionally, lack of Nlgn4 in the KO needs to be confirmed.

2. Statistical rigor: Figure 4 demonstrating increased PPI, is based on n=16-17 animals, while all other data is typically in the n=8-10 range. Figure 5 for example, seems to suggest there are differences in action potential firing patterns between genotypes, but are discarded as being minor. Was sample size analysis performed? The authors need to show that the studies with non-significant trends were adequately powered prior to drawing conclusions.

3. The description of trends as conclusive: In several instances, results that approach significance (p = 0.15 for population spike amplitude as a proxy for granule cell excitability, p = 0.09 for the minimum stimulus intensity to produce a population spike, p = 0.08 for granule cell input resistance, p = 0.12 for granule cell probability to enter depolarization block, p = 0.06 for adaptation at maximum firing frequency, p = 0.13 for stimulated EPSC/IPSC in granule cells) are elsewhere described as slight changes [Introduction line 72, Results line 313, 355, 415, Discussion line 576]. This can be misleading to readers who may not be keeping close track of what was significant, and it would increase clarity to describe granule cells as ‘trending’ toward increased excitability, for example.

Sometimes the ‘slight’ qualifier was omitted: in the results [line 364-365] KO granule cell excitability in vivo was described as decreased based on p = 0.15 [line 300]. The granule cell firing frequencies were described as higher in WT [line 397] and reduced in KO [line 403] but the p value given for maximum firing frequency was 0.4 [line 400]. In the discussion conclusion the intrinsic excitability of granule cells in slice cultures was described as altered [line 711-712], but for input resistance p = 0.08 [line378].

4. The authors conclude there is increased inhibition to granule cells in adult Nlgn4 KOs, but a finding of increased spontaneous or miniature IPSC frequency or amplitude in granule cells from adult KOs would be a direct confirmation. Given the cited roles of Nlgn1 for excitatory inputs onto dentate interneurons, and of Nlgn2 for inhibitory inputs onto granule cells, and of Nlgn4 for inhibitory inputs onto CA3 pyramids, it seems reasonable to consider a role for Nlgn4 at synapses onto dentate interneurons. Expression in interneurons might be consistent with the relatively weak expression of Nlgn4 cited [lines 691-695]. For example, if Nlgn4 has a role in assembly/maintenance of inhibitory synapses onto dentate interneurons, in the KO inhibitory inputs to interneurons maybe reduced thereby increasing downstream inhibition of granule cells. Nlgn4 could be postsynaptic to the similar perisomatic interneuron types in dentate interneurons as CA3 pyramidal cells [line 592-594]. Minimally, this needs to be discussed.

5. Figure 9 presents model for their findings indicating a change in E/I balance. However, the explicit examination of E/I ratio did not identify a difference. While the figure alludes to the in vivo PPI increase, the schematic as depicted is confusing/misleading.

Minor comments

6. [line 399] verify if WT should be KO.

7. Why are Tables 2 and 3 (and their statistics) set up to compare Control vs. TTX for mEPSC amplitude but to compare WT vs KO for mEPSC frequency? The format for Table 2 may be more appropriate to compare the effect of genotype on homeostatic plasticity. Ideally, these comparisons need to be a two-way ANOVA for effect on genotype and TTX as well as interaction.

8. A few errant spaces/periods: lines 510, 525, 658, 996, 1006
